# Temporal changes in the transcriptome profile of *Macrobrachium rosenbergii* in response to decapod iridescent virus 1 infection

**DOI:** 10.3389/fimmu.2025.1575476

**Published:** 2025-04-10

**Authors:** Jingwen Hao, Yukun Jie, Zhibin Lu, Tiantian Ye, Jilun Meng, Cui Liu, Junjun Yan, Yutong Zheng, Zaijie Dong, Zhimin Gu

**Affiliations:** ^1^ Xianghu Laboratory, Hangzhou, China; ^2^ Wuxi Fisheries College, Nanjing Agricultural University, Wuxi, China

**Keywords:** decapod iridescent virus 1, *Macrobrachium rosenbergii*, temporal changes, transcriptome, immune response

## Abstract

The farming of *Macrobrachium rosenbergii* faces significant challenges due to infections caused by Decapod iridovirus 1 (DIV1). To gain deeper insights into the dynamic immune regulatory processes of *M. rosenbergii* in response to DIV1 infection, RNA sequencing (RNA-seq) was employed to profile the transcriptome in the hepatopancreas at 24, 48, 72, and 96 hours post-infection (hpi). Time-course analysis revealed 3,339 differentially expressed genes (DEGs), which exhibited distinct expression patterns across various stages of infection. At 24 hpi and 48 hpi, the top 20 enriched pathways included 3 immunity-related pathways (Lysosome, Phagosome, C-type lectin receptor signaling) and 7 metabolism-related pathways at 24 hpi, and 5 metabolism-related pathways at 48 hpi. In contrast, in the later stages of infection (72 hpi), 13 of the top 17 enriched pathways associated with DEGs were metabolism-related, including those involved in antioxidant defense, such as the Peroxisome, Cysteine and methionine metabolism, and Glutathione metabolism. At 96 hpi, pathways related to ECM-receptor interaction, Purine metabolism, and Lysosome were significantly enriched. Among the DEGs, a total of 16 genes were consistently identified across all time points, with 14 of these genes, including *alpha-2-macroglobulin-like*, *alpha-amylase 1-like*, *putative aldolase class 2 protein PA3430*, *platelet-derived growth factor subunit B-like*, *serum amyloid A-5 protein-like*, *phenoloxidase-activating enzyme-like*, *pantetheinase-like*, and *perlucin-like protein*, demonstrating sustained upregulation at all time points. In contrast, the gene encoding rhodanese domain-containing protein CG4456-like was consistantly downregulated. Additionally, weighted gene co-expression network analysis (WGCNA) indicated several hub genes that were tightly connected to intercellular communication, such as *innexin shaking-B-like* and *innexin inx3-like*, and *endochitinase A1-like*. The gene expression changes varied over time, exhibiting a dynamic, time-dependent pattern that underscores the complexity of host-pathogen interactions. These results provide new insights into the cellular mechanisms influenced by DIV1 throughout the infection process, offering valuable knowledge for developing virus control strategies in shrimp aquaculture.

## 1 Introduction

*Macrobrachium rosenbergii* (*M. rosenbergii*), also known as the giant freshwater prawn, is a highly valuable aquaculture species distributed across tropical and subtropical regions (1). As one of the largest freshwater shrimp species, it can grow up to 30 cm in length and exceed 500 g in weight. Due to its high-quality meat, nutritional value, and strong market demand, *M. rosenbergii* plays a significant role in global aquaculture (2). China, as the world’s leading producer, has experienced rapid growth in *M. rosenbergii* production (3). According to the China Fishery Statistical Yearbook, the country’s production of *M. rosenbergii* has steadily increased since 2018, accounting for over 50% of global output by 2021 (1). In 2023, China achieved a production of 196,374 tons, an increase of 10.42% compared to the previous year (4). However, the expansion of aquaculture has introduced challenges, particularly disease outbreaks such as those caused by Decapod iridescent virus 1 (DIV1) (5), which have led to mortality rates exceeding 80% in infected populations (6). DIV1 is a newly identified viral strain in crustaceans, characterized by its icosahedral structure, a diameter of approximately 150 nm, and a double-stranded DNA genome spanning about 166 kb (7). In 2019, the International Committee on Taxonomy of Viruses officially classified the previously identified SHIV (8) and CQIV (9, 10) isolates as a new species, Decapod iridescent virus 1 (DIV1), within the newly established genus *Decapodiridovirus*, part of the subfamily *Betairidovirinae*. Current research on DIV1 has primarily focused on its genomic structure, pathogenicity (5, 9), detection methods (11, 12), clinical symptoms (5, 13), and host histopathology (5, 13). Despite these advances, there is a lack of research on the immune responses of *M. rosenbergii* to DIV1 infection.

Advancements in RNA sequencing technology have significantly advanced our understanding of virus-host interactions and immune mechanisms in crustaceans during viral infections. For example, transcriptomic studies in *Penaeus monodon* have identified key immune-related pathways, such as PI3K-Akt, Toll and Imd, NF-kappa B, and MAPK signaling, in response to DIV1 infection (14). Similar studies on other species, including *Litopenaeus vannamei*, *Marsupenaeus japonicus*, *Fenneropenaeus merguiensis*, and *Cherax quadricarinatus*, have highlighted the immune responses involving Lysosome and Phagosome, which play essential roles in defending against viral infections (15, 16). However, studies on *M. rosenbergii* remain limited, with only one study employing quantitative real-time polymerase chain reaction (qRT-PCR) to assess the expression of immune-related genes, such as prophenoloxidase (proPO) and anti-lipopolysaccharide factors (ALFs), following DIV1 infection (17). The above-mentioned study provides a narrow view of the immune response, underscoring the need for a more comprehensive transcriptomic approach to fully understand the dynamic immune response in *M. rosenbergii* during DIV1 infection. This study aims to address this gap by performing a time-series transcriptomic analysis to examine the temporal changes in gene expression and immune pathways during different stages of DIV1 infection in *M. rosenbergii*.

Weighted gene co-expression network analysis (WGCNA) is a systems biology method for analyzing large-scale gene expression data (18), identifying hub genes, and uncovering key regulatory networks. By integrating WGCNA with differentially expressed gene (DEG) analysis, researchers can explore molecular responses and pathways involved in *M. rosenbergii*’s response to DIV1 infection over time. WGCNA constructs gene co-expression networks by grouping genes with similar expression patterns across samples, using correlation coefficients to measure relationships. Co-expression modules are then linked to phenotypic traits, such as disease status, to reveal key genes and their roles in biological processes.

Like other crustaceans, *M. rosenbergii* relies on its innate immune system for pathogen defense (19). The hepatopancreas plays a critical role in immune responses and digestion in crustaceans (20–22), and recent studies have further demonstrated that it is one of the major target organs for DIV1 infection (17, 23, 24). This study performed a time-series transcriptomic analysis of the hepatopancreas in *M. rosenbergii* infected with DIV1 at 24, 48, 72, and 96 hpi. DEGs were analyzed using Gene Ontology (GO) and Kyoto Encyclopedia of Genes and Genomes (KEGG) enrichment analyses, providing insights into the host’s dynamic molecular responses. A weighted gene co-expression network was constructed to identify modules associated with infection time and pinpoint key hub genes. The research results reveal the temporal immune response dynamics and mechanisms underlying the interactions between DIV1 and *M. rosenbergii*, providing insights into the immune processes and offering guidance for virus control strategies.

## 2 Materials and methods

### 2.1 Experimental animals

Giant river prawns (*Macrobrachium rosenbergii*) with an average length of 6.5 cm were sourced from a farm in Jiaxing City, Zhejiang Province, and used throughout the study. Prior to the commencement of experimental procedures, five prawns were randomly selected for pathogen screening, including DIV1, infectious precocity virus (IPV), white spot syndrome virus (WSSV), infectious hypodermal and hematopoietic necrosis virus (IHHNV), *Vibrio parahaemolyticus* (VpAHPND), and *Enterocytozoon hepatopenaei* (EHP). Pathogen detection was performed via qRT-PCR method following the protocol described in section 2.2, using the specific primers listed in **Table 1**. The prawns were acclimated in a controlled laboratory environment for 7 days before the start of the experiments. During this acclimation period, they were kept at room temperature and fed a commercial diet, at approximately 3% of their body weight daily. Any uneaten feed and fecal matter were removed daily to ensure the maintenance of water quality.

**Table 1:** qRT-PCR detection primers and probes.

Primer Name	Primer and Probe Sequence (5′–3′)	Reference
DIV1-qF	AGGAGAGGGAAATAACGGGAAAAC	(12)
DIV1-qR	CGTCAGCATTTGGTTCATCCATG	
Probe	FAM-CTGCCCATCTAACACCATCTCCCGCCC-TAMRA	
IPV-qF	AGGAGAGGGTTTTGGCTTG	(90)
IPV-qR	CTGGATTGGAAGGGAACTCTG	
Probe	FAM-GAAGATGTCATCGTCCCAGAGTT-TAMRA	
WSSV-qF	TGAGGTTGGATCAGGCTACTTC	(91)
WSSV-qR	CCGCATCTTCTTCCTTCATCTG	
Probe	FAM-CAAGTACCCAGGCCCAGTGTCATACGTT-TAMRA	
IHHNV-qF	TACTCCGGACACCCAACCA	(92)
IHHNV-qR	GGCTCTGGCAGCAAAGGTAA	
Probe	FAM-ACCAGACATAGAGCTACAATCCTCGCCTATTTG-TAMRA	
AHPND-qF	TTGGACTGTCGAACCAAACG	(93)
AHPND-qR	GCACCCCATTGGTATTGAATG	
Probe	FAM-AGACAGCAAACATACACCTATCATCCCGGA-TAMRA	
EHP-qF	AGTAAACTATGCCGACAA	(94)
EHP-qR	AATTAAGCAGCACAATCC	
Probe	FAM-TCCTGGTAGTGTCCTTCCGT-TAMRA	

### 2.2 Isolation and preparation of DIV1

The DIV1-ZH strain (GenBank accession number PQ724921) was isolated from tissues of naturally infected *Litopenaeus vannamei* in Zhuhai, Guangdong Province, China. For sample preparation, hepatopancreas tissues from diseased shrimp were homogenized in phosphate-buffered saline (PBS) at a 1:10 ratio using a sterile, enzyme-free glass grinding tube. The tube was placed in an ice-water bath and gently ground to form a homogenate. The homogenate was then subjected to sequential centrifugation: first at 3,000 rpm for 20 minutes at 4°C, followed by a second centrifugation at 8,000 rpm for 25 minutes at 4°C. The final supernatant was filtered through a 0.22 µm membrane filter and stored at –80°C. DIV1 DNA was extracted using the Genomic DNA/RNA Extraction Kit for Aquatic Animal Pathogens (FAST) (DHelix, Guangzhou, China). The qRT-PCR reaction mixture included 10 μL of AceQ^®^ Universal U^+^ Probe Master Mix V2 (Vazyme Biotech Co., Ltd, Nanjing, China), 0.4 μL of DIV1-qF (10 μM), 0.4 μL of DIV1-qR (10 μM), 0.2 μL of TaqMan Probe (10 μM), 1 μL of template DNA, and 8 μL of ddH_2_O. The primers and probe are listed in **Table 1**. The amplification protocol consisted of an initial denaturation at 37°C for 2 minutes, followed by 95°C for 5 minutes, and then 40 cycles of 95°C for 10 seconds and 60°C for 30 seconds. qRT-PCR was performed on the Applied Biosystems™ QuantStudio™ 3 (Thermo Fisher Scientific, USA). DIV1 copy numbers were determined based on a standard curve constructed in our laboratory, as well as DNA concentration and the cycle threshold (Ct) value.

### 2.3 Experimental design and sampling of *M. rosenbergii*

The randomly selected shrimps tested negative for DIV1, IPV, WSSV, IHHNV, VpAHPND, and EHP, indicating the shrimps were suitable for subsequent experiments. The experiment was conducted in a completely randomized design with two groups. Each treatment consisted of 100 prawns housed in 120 L glass tanks, with a stocking density of 25 individuals per tank. Each shrimp in the control group was injected with 100 μL of sterile PBS. The shrimps in the infected group were each given with 100 µL DIV1 inoculum at a concentration of 1.30 × 10^8^ copies/mL, which corresponds to the semi-lethal concentration at 72 hpi. Water was replaced daily at a rate of 50% to maintain optimal conditions. Animals were fed twice daily, with adjustments made based on observed consumption rates. Hepatopancreas tissues were collected from each group at 24, 48, 72, and 96 hpi for RNA extraction. Four replicate samples were collected from each group at each time point.

### 2.4 RNA extraction, library construction, and transcriptome sequencing

Total RNA was extracted from the hepatopancreas using TRIzol^®^ Reagent (Qiagen, Germany) in accordance with the manufacturer’s guidelines. The concentration and purity of the extracted RNA were detected using a Nanodrop 2000 (Thermo Fisher Scientific, CA, USA). RNA integrity was evaluated by agarose gel electrophoresis (DYY-6C, China), and the RNA Quality Number (RQN) value was determined using an Agilent5300 (Agilent, USA). Only high-quality RNA samples with the following criteria were used for sequencing library construction: OD260/280 = 1.8–2.2, OD260/230 ≥ 2.0, RQN ≥ 6.5, 28S:18S ≥ 1.0, and RNA yield > 1 μg. The RNA-seq transcriptome library of the hepatopancreas was constructed with the Illumina^®^ Stranded mRNA Prep, Ligation (San Diego, CA) with 1μg of total RNA. Briefly, messenger RNA (mRNA) was enriched using the polyA selection technique with oligo (dT) beads and then fragmented. Double-stranded cDNA was then generated using random hexamer primers. The generated cDNA underwent end-repair, phosphorylation, and adaptor insertion in accordance with the library creation methodology. The libraries were size-selected for target cDNA fragments of approximately 300 bp using magnetic beads, followed by PCR amplification for 15 cycles. Following quantification by Qubit 4.0 (Thermo Fisher Scientific, USA), the sequencing library was executed on the NovaSeq X Plus platform (PE150) (Illumina, USA) with the NovaSeq Reagent Kit (Illumina, USA).

### 2.5 Data processing and bioinformatics analysis

The raw paired-end reads were trimmed and subjected to quality control using fastp (25). Raw sequencing data were filtered to obtain high-quality clean data by removing adapter sequences, reads without insert fragments, trimming low-quality bases (quality < 20) at the 3’ end, discarding reads with a remaining quality < 10, and eliminating reads with > 10% N content or those shorter than 20 bp after trimming. HISAT2 (26) was used in orientation mode to map the quality-controlled clean reads to the *M. rosenbergii* reference genome (GCF_040412425.1, https://www.ncbi.nlm.nih.gov/datasets/genome/GCF_040412425.1/), generating mapped data for transcript assembly and expression quantification. The mapped reads from each sample were assembled using StringTie (27). The gene expression was quantified based on read counts in the genome regions, and expression levels were normalized using the transcripts per million reads (TPM) method with RSEM (28). Differential expression analysis among samples was conducted via DESeq2 (29), and the significantly DEGs were identified by false discovery rate (FDR) < 0.05 and |log2FC| ≧ 1.

### 2.6 Function annotations and enrichment analysis

Gene function annotation was performed using several databases, including Gene Ontology (GO), KEGG Orthology (KO), Evolutionary Genealogy of Genes: Non-supervised Orthologous Groups (EggNOG), NCBI non-redundant protein sequences (Nr), a curated protein sequence database (Swiss-Prot), and Protein families (Pfam). GO enrichment analysis was conducted to categorize gene products by mapping unigenes to functional categories. KEGG enrichment analysis was performed to map unigenes onto known signaling pathways. GO and KEGG functional enrichment of DEGs were carried out using their respective databases, respectively. A hypergeometric test with a threshold of *p* < 0.05 was applied to identify significantly enriched GO terms and KEGG pathways.

### 2.7 Weighted gene co-expression network analysis

WGCNA is a widely used algorithm for constructing gene co-expression networks by grouping genes with similar expression patterns across samples. Co-expression relationships are quantified using correlation coefficients of gene expression levels. To ensure analytical accuracy, we initially preprocessed 31,758 reference genes to reduce noise. Genes with expression levels below 1 and coefficients of variation under 0.1 were excluded, resulting in a dataset of 7,544 genes for further analysis. The network type was set to “signed” to preserve regulatory directionality, with a soft-threshold power (β) of 0.7 applied to measure gene correlations. Modules were defined with a minimum size of 30 genes, with a correlation coefficient of at least 0.3 between module members and eigengenes. To refine the analysis, modules with a clustering distance below 0.25 were merged, enhancing the interpretability of the co-expression networks.

### 2.8 Validation of gene expression by qRT-PCR

To validate the gene expression identified via RNA sequencing, qRT-PCR was performed using the same RNA samples. The expression levels of eight genes, namely LOC136838548 (*PDGF*, platelet-derived growth factor subunit B-like), LOC136838681 (*FTCD*, formimidoyltransferase-cyclodeaminase-like), LOC136838726 (*ALF3*, anti-lipopolysaccharide factor-like), LOC136826554 (*α2M*, alpha-2-macroglobulin-like), LOC136831547 (*hsp90*, heat shock protein 90), LOC136826055 (*NRFP6*, nose resistant to fluoxetine protein 6-like), LOC136848898 (*CA4*, Carbonic anhydrase), and LOC136843976 (*BBOX1*, gamma-butyrobetaine dioxygenase-like) were analyzed using *β-actin* (LOC136855692) as the internal control for normalization. These genes are related to cell signaling and proliferation regulation, immune defense, and energy metabolism. Each sample was analyzed in triplicate. Primers specific to the target genes were designed with Primer 5.0, and their sequences are provided in **Table 2**. Gene expression changes were calculated using the 2^−ΔΔCt^ method (30), and the results were represented as log2fold changes for visual presentation.

**Table 2:** Primers used for qRT-PCR.

Primers	Gene name	Gene description	Sequence (5′–3′)	Product Size (bp)	Source
PDGF-qF	LOC136838548	platelet-derived growth factor subunit B-like	AAATCGGTGCCAAGAGCC	118	This study
PDGF-qR			CATCATCCAGCAATATCCATCA		
FTCD-qF	LOC136838681	formimidoyltransferase-cyclodeaminase-like	GCCACTGTGACAGGTGTTCG	253	This study
FTCD-qR			CGTCTTTCTTAGCCTCTTCGTAT		
ALF3-qF	LOC136838726	anti-lipopolysaccharide factor-like	AACATCATCGGCACTGCAAAG	168	(17)
ALF3-qR			ATTCCTCCCACAACTGATGGC		
2α2M_qF	LOC136826554	alpha-2-macroglobulin-like	GCACAGGGGAGGAGTTTGTT	150	(17)
2α2M_qR			AAGCAGGAGAACCATCTGGC		
Hsp90-qF	LOC136831547	heat shock protein 90	CTGACAAGGTCACAGTCGTT	179	This study
Hsp90-qR			CTTGATACGACGTTCCTCCA		
NRFP6-qF	LOC136826055	nose resistant to fluoxetine protein 6-like	TTTCTCCTACGACGAATCTGC	158	This study
NRFP6-qR			TGCTATGTTACCCTCCACGAC		
CA4-qF	LOC136848898	Carbonic anhydrase	GGTTATCGCAGTCTCCTATTGA	142	This study
CA4-qR			GCTGTGACTTGAGCCGTGT		
BBOX1-qF	LOC136843976	gamma-butyrobetaine dioxygenase-like	TGCGTGAAGCAGCACATTG	119	This study
BBOX1-qR			GAGTCGGTGGTGGTCAGGAT		
β-actin-qF	LOC136855692	actin	TGTTCGAGACCTTCAACACCC	158	(17)
β-actin-qR			GGAGGATGGCATGAGGAAGTG		

## 3 Results

### 3.1 Transcripome sequencing data

A high-throughput second-generation sequencing platform is capable of generating billions of reads in a single run, but such a large volume of data does not provide insight into the quality of individual reads. By applying statistical methods for sequence analysis and quality control, the overall quality of the library construction and sequencing could be assessed from a macro perspective. In this study, transcriptome analysis was performed on 32 samples, yielding a total of 211.01 Gb of clean data. Each sample generated 5.94 Gb of clean data, with the Q30 base percentage exceeding 96.06% (**Table 3**). The mapping rates of clean reads for each sample to the reference genome ranged from 92.32% to 94.86% (**Table 3**).

**Table 3:** Statistics of sequencing data.

Sample	Raw reads	Clean reads	Total mapped	Q20 (%)	Q30 (%)	GC content (%)
C24_1	46778122	46414150	43736814 (94.23%)	98.91	96.42	44.42
C24_2	44114590	43845970	41585044 (94.84%)	98.91	96.42	43.29
C24_3	45873142	45503692	42586531 (93.59%)	98.88	96.39	43.15
C24_4	44781638	44439422	41427389 (93.22%)	98.78	96.06	42.55
T24_1	46803366	46461488	43786211 (94.24%)	98.88	96.32	45.24
T24_2	42288640	42012508	39854713 (94.86%)	98.9	96.38	43.97
T24_3	45147146	44860486	42433405 (94.59%)	98.88	96.31	44.05
T24_4	44470498	44150916	41465002 (93.92%)	98.87	96.31	43.8
C48_1	40838866	40546066	37720775 (93.03%)	98.82	96.16	43.65
C48_2	41050334	40757928	37885122 (92.95%)	98.8	96.13	42.81
C48_3	47188412	46852302	44272509 (94.49%)	98.91	96.41	44.57
C48_4	46329652	45936420	42712881 (92.98%)	98.86	96.32	43.1
T48_1	45372942	45059368	42601942 (94.55%)	98.89	96.34	45.18
T48_2	41033390	40732070	38407445 (94.29%)	98.89	96.35	44.27
T48_3	45600562	45264350	42818583 (94.6%)	98.88	96.34	45.47
T48_4	43779166	43412042	41074790 (94.62%)	98.84	96.19	44.53
C72_1	41093864	40778958	38240842 (93.78%)	98.88	96.33	43.94
C72_2	47212108	46824998	44092234 (94.16%)	98.9	96.38	44.37
C72_3	50250582	49898778	46593201 (93.38%)	98.88	96.35	44.39
C72_4	48596580	48257816	45403630 (94.09%)	98.9	96.37	44.79
T72_1	41666194	41361980	38867311 (93.97%)	98.89	96.39	44.16
T72_2	45224102	44889606	42066079 (93.71%)	98.86	96.27	44.61
T72_3	45347008	44915290	42014884 (93.54%)	98.86	96.29	43.89
T72_4	45204726	44826272	41828180 (93.31%)	98.89	96.4	44.01
C96_1	50708122	50296638	47578492 (94.6%)	98.87	96.31	43.42
C96_2	43384330	43092534	40701100 (94.45%)	98.9	96.41	43.44
C96_3	40215644	39919666	36855319 (92.32%)	98.86	96.31	44.75
C96_4	40726642	40432948	38076936 (94.17%)	98.88	96.33	45.21
T96_1	41865214	41573004	38866264 (93.49%)	98.89	96.38	44.16
T96_2	45754972	45394024	42312117 (93.21%)	98.88	96.35	44.83
T96_3	48775902	48400512	45231733 (93.45%)	98.86	96.28	44.38
T96_4	43588688	43268978	39954542 (92.34%)	98.81	96.18	44.22

(1) Sample: Sample name; (2) Raw reads: The total number of raw sequencing reads (reads represent sequencing fragments, with each read corresponding to one fragment); (3) Raw bases: The total amount of raw sequencing data (i.e., the number of raw reads multiplied by the read length); (4) Clean reads: The total number of sequencing reads after quality control; (5) Clean bases: The total amount of sequencing data after quality control (i.e., the number of clean reads multiplied by the read length); (6) Error rate (%): The average error rate of sequencing bases corresponding to the quality-controlled data, typically below 0.1%; (7) Q20 (%), Q30 (%): Quality assessment of the quality-controlled sequencing data. Q20 and Q30 refer to the percentage of bases with sequencing quality greater than 99% and 99.9%, respectively, in the total bases.

The distribution of reads mapped to different regions of the reference genome was analyzed, including coding sequences (CDS), introns, intergenic regions, and 5’ and 3’ untranslated regions (UTRs). Among the 32 samples, the highest proportion of mapped reads was located in the CDS region (56.33%-70.84%), followed by the 3’ UTR (12.83%-19.9%), the 5’ UTR (8.7%-12.1%), introns (4.08%-6.89%), and intergenic regions (1.58%-3.9%) (**Supplementary Table S1**). The number of sequences mapped to each chromosome was analyzed to provide an overview of the distribution of sequencing reads across chromosomes. The results showed that the mapped reads were distributed similarly across the chromosomes in both the control and DIV1-infected groups. In the control group, the largest numbers of mapped reads were located on chromosomes NC_089788.1, NC_089753.1, NC_089741.1, NC_089752.1, NC_089764.1, and NC_089795.1. In the DIV1-infected group, the highest numbers of mapped reads were found on chromosomes NC_089753.1, NC_089788.1, NC_089741.1, NC_089752.1, NC_089764.1, and NC_089795.1 (**Supplementary Figure S1**).

### 3.2 Transcript length distribution and functional annotation of expressed genes

The length distribution of the transcripts is shown in **Supplementary Figure S2A**. The majority of the transcripts (15,376, 19.69%) were between 0 and 1000 nt in length, followed by those in the 1001–2000 nt range (14,424, 18.47%), and 12,780 transcripts (16.37%) fell within the 2001–3000 nt range. A total of 23,491 expressed genes were detected, of which 21,605 were known genes and 1,886 were novel genes. All genes obtained from transcriptome assembly were compared against six major databases (NR, Swiss-Prot, Pfam, EggNOG, GO, and KEGG) to acquire comprehensive functional information. The annotation results for each database were then statistically analyzed. The annotation results revealed the following distribution: GO (13,543 unigenes, 42.64%), KEGG (11,176 unigenes, 35.19%), EggNOG (17,420 unigenes, 54.85%), Nr (22,790 unigenes, 71.76%), Swiss-Prot (14,793 unigenes, 46.58%), and Pfam (16,517 unigenes, 52.01%) (**Supplementary Table S2**). The Nr database contained the highest number of homologous sequences in the assembled genes, with 17,644 genes annotated (**Supplementary Figure S2B**).

### 3.3 Viral genes detected in transcriptomic data

**Figure 1:**
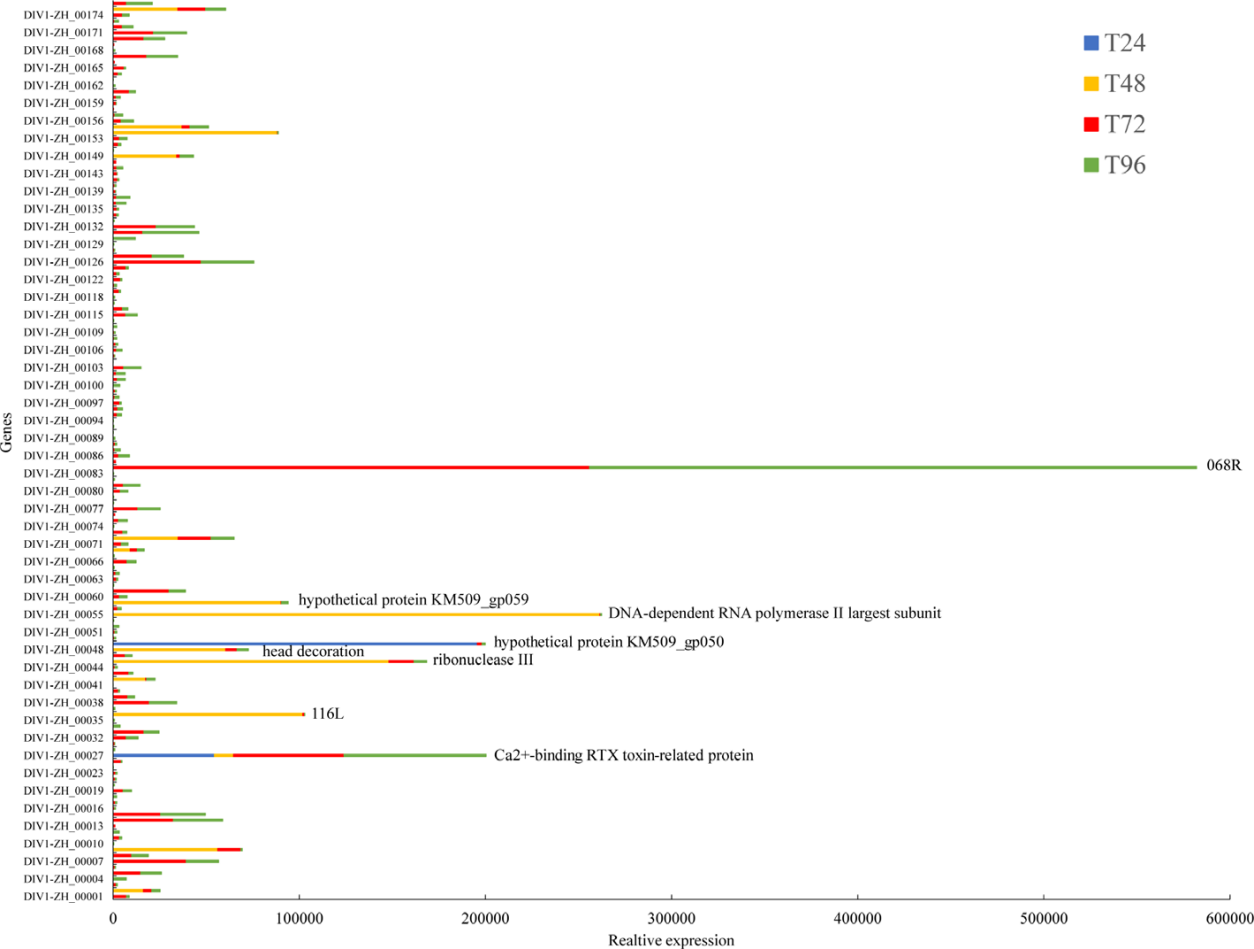
The relative expression levels of viral genes detected in the transcriptomic data at different time points. The x-axis represents the relative expression levels, while the y-axis corresponds to different DIV1 genes. The color of the bars indicates different time points, with taller bars representing a larger number of viral sequences.

In the preliminary phase, our laboratory performed whole-genome sequencing of DIV1, followed by genome assembly and annotation. The results revealed that this strain comprises 176 open reading frames (ORFs). Subsequently, we aligned the quality-controlled RNA-seq data with the DIV1 genome (accession number: PQ724921) to validate the presence of viral transcripts and assess their relative abundance. The analysis revealed that none of the control group transcriptome samples from the four time points mapped to viral transcript sequences. In contrast, the transcriptome samples from the DIV1-infected group identified 153 DIV1-encoded gene sequences, with distinct temporal variations observed (**Supplementary Table S3**). Although the functional roles of most viral alleles remain undetermined, we successfully identified several known genes, including the Ca²^+^-binding RTX toxin-related protein, DNA-dependent RNA polymerase II largest subunit, ribonuclease III, and head decoration (**Figure 1**).

### 3.4 Principal component analysis of expression levels between samples

**Figure 2:**
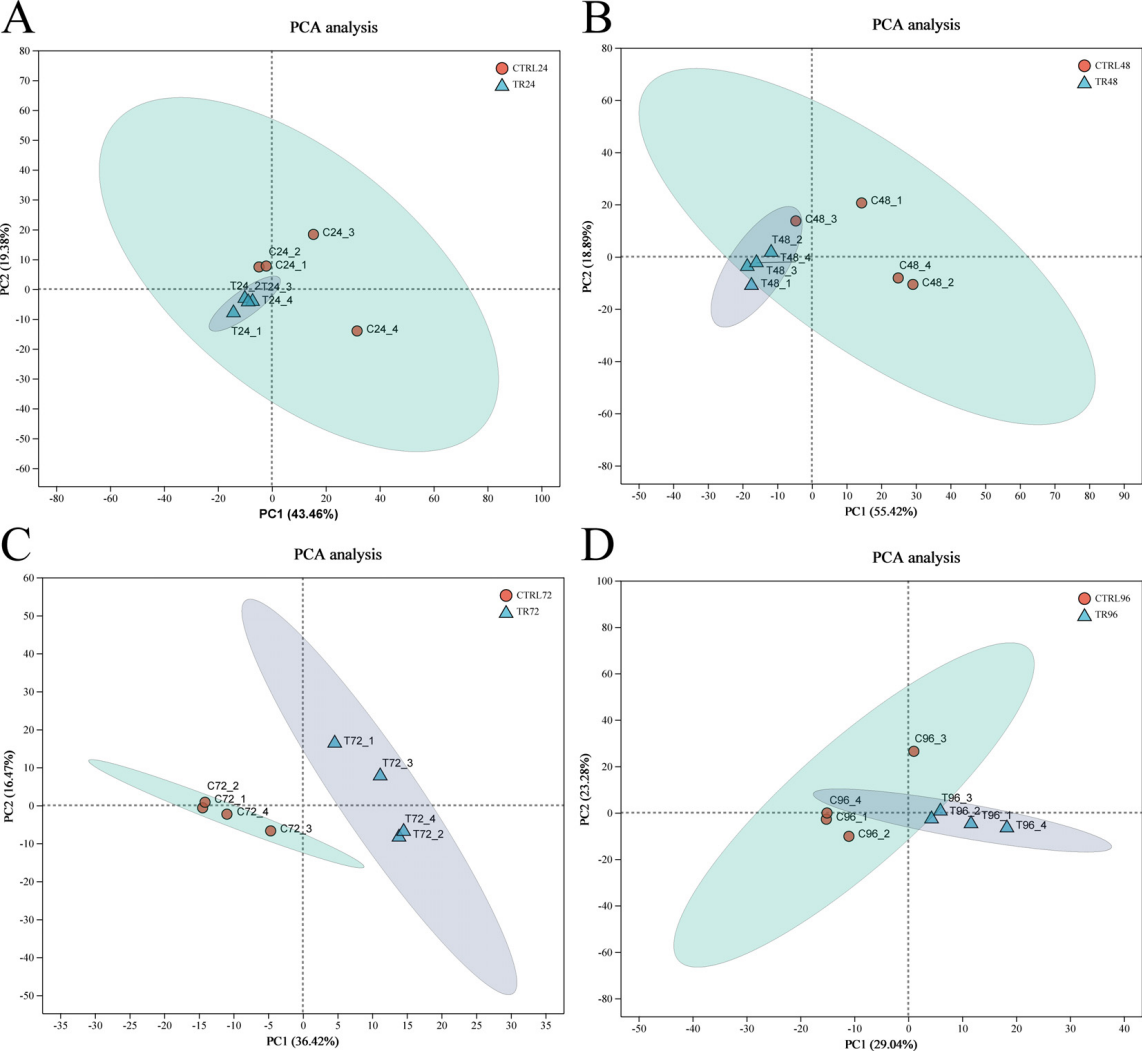
Principal component analysis plots. **(A-D)** represent the PCA of gene expression between DIV1-infected samples at 24, 48, 72, and 96 hpi and their corresponding control samples. After dimensionality reduction, the plot shows relative coordinate points along the principal components, where the distance between sample points reflects their similarity. Closer points indicate higher similarity between the samples. The x-axis represents the contribution of principal component 1 (PC1) to sample differentiation, while the y-axis represents the contribution of principal component 2 (PC2) to sample differentiation.

This study utilized transcriptomic data from different time points (24, 48, 72, and 96 hpi) after DIV1 infection to analyze its impact on gene expression. Principal component analysis (PCA) was performed based on the expression matrix to explore the expression differences between the samples. The primary aim was to reduce the data complexity through dimensionality reduction and reveal potential differences between the DIV1-infected samples at different time points and their corresponding controls, as well as similarities among the samples. PCA analysis of the prawn samples showed a significant distinction between the infected and uninfected groups. As shown in **Figure 2**, the first principal component (PC1) accounted for approximately 43.46%, 55.42%, 36.42%, and 29.04% of the variance in the samples at 24, 48, 72, and 96 hpi, respectively. The second principal component (PC2) explained 19.38%, 18.89%, 16.47%, and 23.28% of the variance. The PCA plot clearly demonstrates that the DIV1-infected samples are distinctly separated from their corresponding control samples along both PC1 and PC2. Notably, the infected samples exhibit a significant shift along PC1, indicating a pronounced effect of DIV1 infection on gene expression. Furthermore, the infected samples at different time points display a clustering trend along PC2, suggesting a dynamic shift in gene expression patterns as the infection progresses.

### 3.5 Identification and characterization of the host DEGs

**Figure 3:**
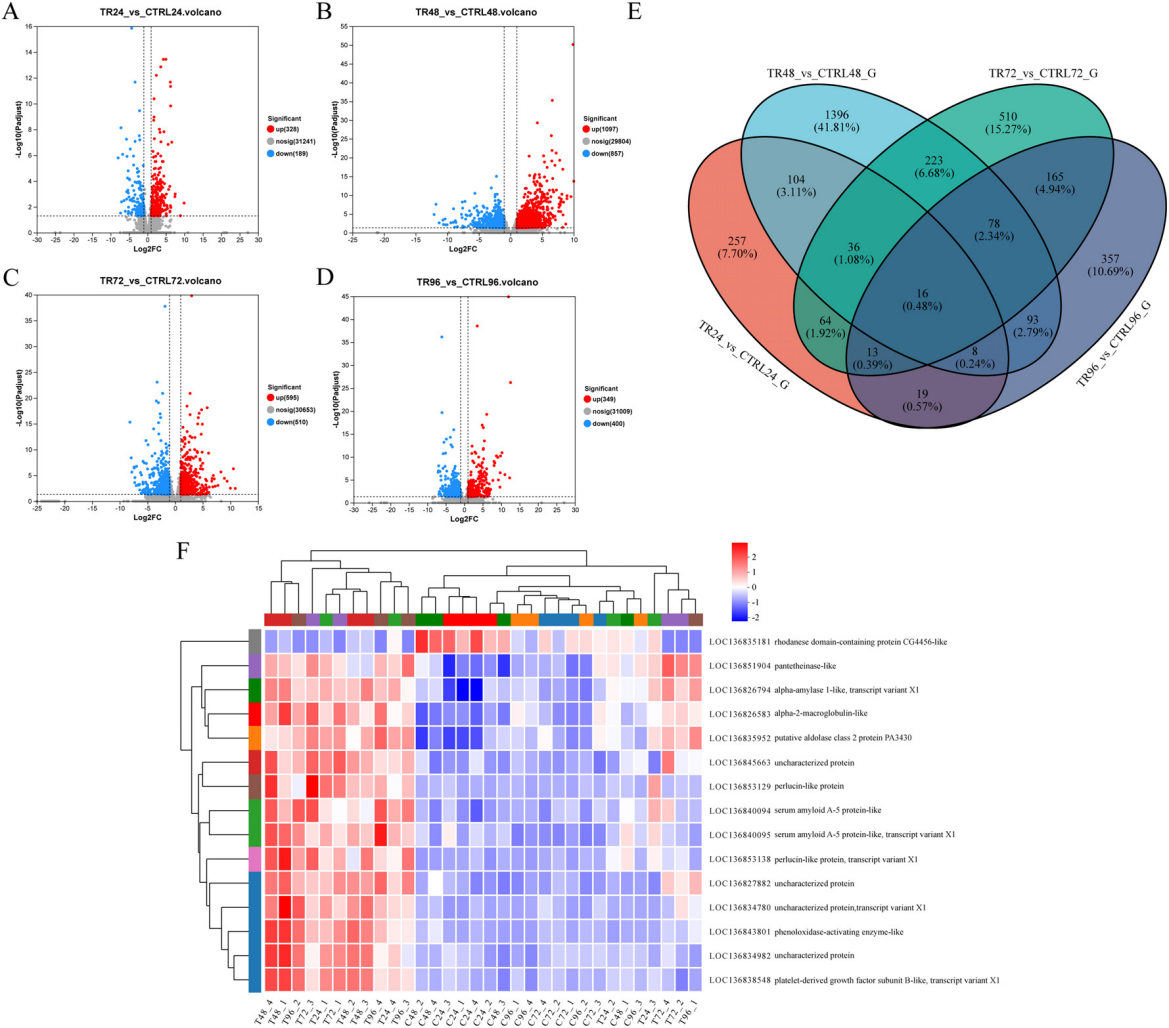
Volcano plot showing DEGs in *M. rosenbergii* between the control and DIV1-infected groups at **(A)** 24 hpi, **(B)** 48 hpi, **(C)** 72 hpi, and **(D)** 96 hpi. The x-axis represents the log2 fold change in gene expression between the two groups of samples, calculated by dividing the expression level of the treatment sample by that of the control sample. The y-axis represents the statistical significance of the expression change, specifically -log10 (p-value), where higher values indicate more significant differences in expression. Each point on the plot corresponds to an individual gene, with red points representing significantly upregulated genes, blue points indicating significantly downregulated genes, and gray points denoting genes with no significant difference. Genes on the left side of the plot exhibit downregulated expression, while those on the right show upregulated expression. Genes closer to the boundary line demonstrate more pronounced and statistically significant changes in expression. **(E)** Venn diagram. The circles in different colors represent different gene sets, and the values indicate the number of common and unique genes between the gene sets. **(F)** A heatmap illustrating the expression dynamics of 14 genes that are consistently upregulated and 1 gene that is consistently downregulated across 4 different sampling time points.

To investigate the DEGs in *M. rosenbergii* associated with DIV1 infection, transcriptome data from both DIV1-infected and control groups were compared. Gene expression levels were quantified using the TPM value for each sample. A total of 517 DEGs were identified in the DIV1-infected groups compared to the control group at 24 hpi, including 328 up-regulated and 189 down-regulated genes. At 48 hpi, 1,954 DEGs were detected, with 1,097 up-regulated and 857 down-regulated genes. At 72 hpi, 1,105 DEGs were identified, including 595 up-regulated and 510 down-regulated genes. Finally, at 96 hpi, 749 DEGs were found, with 349 up-regulated and 400 down-regulated genes (**Figures 3A–D**). The complete repertoire of DEGs across the different sampling points is provided in **Supplementary File S1**. A Venn diagram was used to display the number of genes in each gene set, as well as the overlap between gene sets, identifying common and unique genes between them. As shown in **Figure 3E**, there are 257, 1396, 510, and 357 unique genes in the TR24_*vs*_CTRL24_G, TR48_*vs*_CTRL48_G, TR72_*vs*_CTRL72_G, and TR96_*vs*_CTRL96_G gene sets, respectively. The differentially expressed gene sets at four time points post-infection share 16 genes. Among these, 14 genes exhibited consistently upregulated expression at all four time points, including those encoding alpha-2-macroglobulin-like, alpha-amylase 1-like, putative aldolase class 2 protein PA3430, platelet-derived growth factor subunit B-like, serum amyloid A-5 protein-like (LOC136840094), serum amyloid A-5 protein-like (LOC136840095), phenoloxidase-activating enzyme-like, pantetheinase-like, perlucin-like protein (LOC136853129), and perlucin-like protein (LOC136853138), and four genes encoding uncharacterized proteins. In contrast, the gene encoding rhodanese domain-containing protein CG4456-like shows consistently downregulated expression when comparing the infected group to the control group (**Table 4**). These genes may play important roles in the host’s response to DIV1 infection, and **Figure 3F** shows their expression changes at different time points.

**Table 4:** Genes that were consistently up- and down-regulated throughout all time points in the Infected *vs* Control.

Gene Name	Gene Description
Up-regulated genes at all four time points (Infected *vs* Control)	
LOC136826583	alpha-2-macroglobulin-like
LOC136826794	alpha-amylase 1-like, transcript variant X1
LOC136827882	uncharacterized protein
LOC136834780	uncharacterized protein, transcript variant X1
LOC136834982	uncharacterized protein
LOC136835952	putative aldolase class 2 protein PA3430
LOC136838548	platelet-derived growth factor subunit B-like, transcript variant X1
LOC136840094	serum amyloid A-5 protein-like
LOC136840095	serum amyloid A-5 protein-like, transcript variant X1
LOC136843801	phenoloxidase-activating enzyme-like
LOC136845663	uncharacterized protein
LOC136851904	pantetheinase-like
LOC136853129	perlucin-like protein
LOC136853138	perlucin-like protein, transcript variant X1
Down-regulated genes at all four time points (Infected *vs* Control)	
LOC136835181	rhodanese domain-containing protein CG4456-like

### 3.6 GO enrichment analysis of the host DEGs

**Figure 4:**
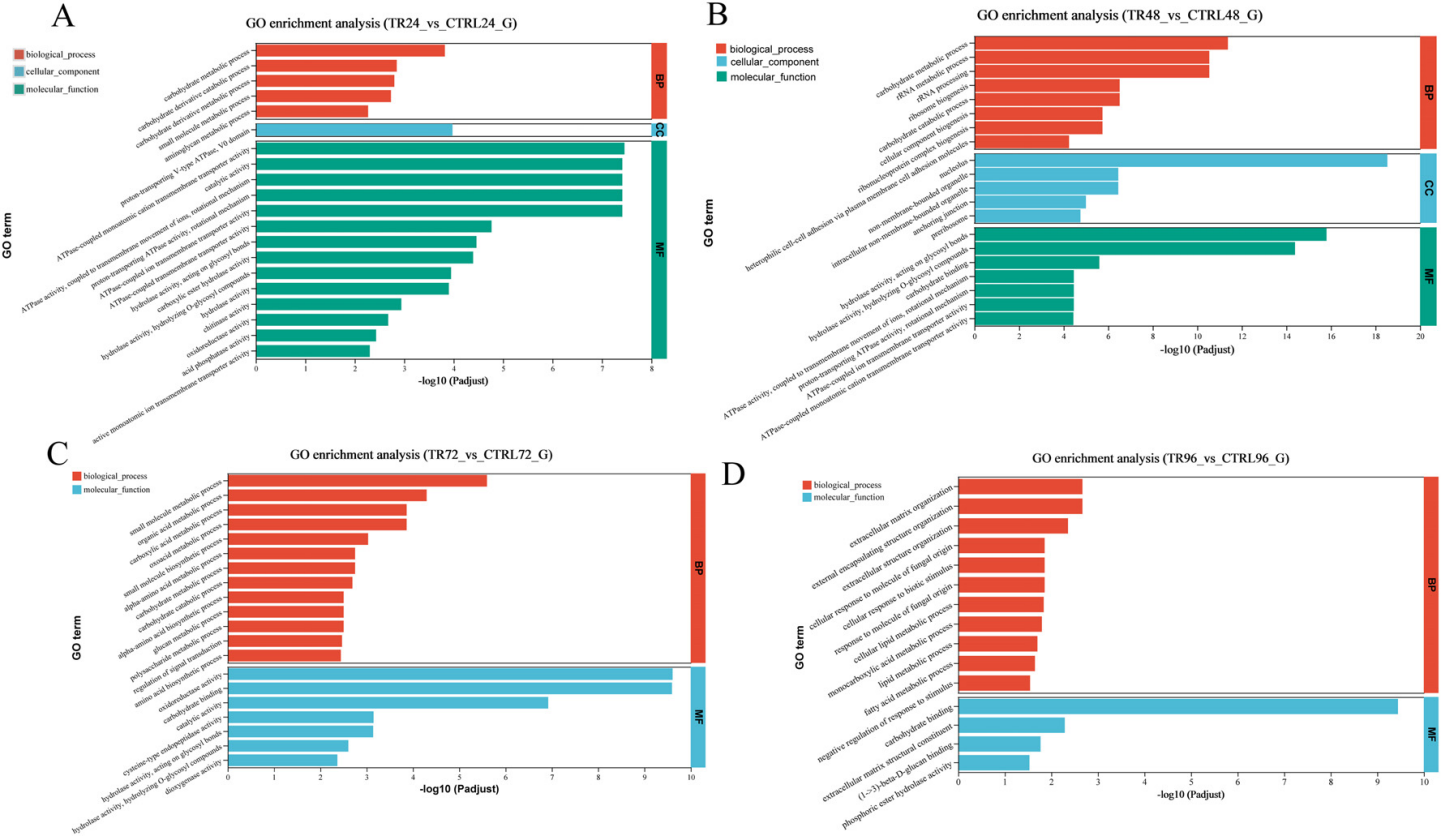
GO enrichment analysis bar plots of DEGs. **(A-D)** show the enrichment of all DEGs between the DIV1-infected and control groups at 24, 48, 72, and 96 hpi, respectively. The analysis was performed using Fisher’s exact test, with a significance threshold set to padjust < 0.05. Each plot displays the top 20 GO terms based on the padjust values. The x-axis indicates the significance level of the enrichment, corresponding to the height of the bars, while the y-axis represents the GO terms. The smaller the Padjust value, the higher the –log10 (Padjust), indicating greater enrichment significance for that particular GO term. The three colors represent the three main categories: biological processes (BP), cellular components (CC), and molecular functions (MF).

To further assess the biological functions, all DEGs were mapped to terms in the GO databases. The top 20 GO terms affected by DIV1 infection at each time point are presented in **Figure 4**. At 24 hpi, DEGs were significantly enriched in GO terms related to ion transport and energy metabolism, such as “ATPase-coupled monoatomic cation transmembrane transporter activity” and “proton-transporting ATPase activity, rotational mechanism.” These terms may reflect the host cell’s early response to viral invasion by regulating ion concentration and cellular energy metabolism. Additionally, the enrichment of terms like “hydrolase activity, acting on glycosyl bonds” and “chitinase activity” suggests that the host may activate hydrolytic enzymes to defend against the virus, particularly enhancing immune responses through carbohydrate metabolism. At 48 hpi, enriched GO terms like “rRNA metabolic process” and “ribosome biogenesis” indicate that the host cell may enhance protein synthesis to support immune responses and cellular repair. At the same time, terms such as “carbohydrate binding” and “ATPase activity, coupled to transmembrane movement of ions” suggest that the host optimizes cell function to cope with the ongoing infection by regulating carbohydrate metabolism and ion transport. At 72 hpi, enriched terms such as “oxidoreductase activity” and “regulation of signal transduction” indicate that the host cell modulates immune responses through redox reactions and signaling pathways. The further enrichment of terms like “carbohydrate metabolic process” and “glucan metabolic process” suggests that the host strengthens immune responses and viral clearance by regulating carbohydrate and polysaccharide metabolism. At 96 hpi, DEGs were enriched in terms like “extracellular matrix organization” and “cellular response to biotic stimulus,” showing that the host cell further promotes viral defense by regulating extracellular matrix organization and responding to biological stimuli. Additionally, the enrichment of terms such as “lipid metabolic process” and “fatty acid metabolic process” suggests that the host strengthens immune defenses through lipid metabolism, regulating membrane structure and signaling to optimize antiviral responses. In conclusion, DIV1 infection triggers the host to coordinate metabolic changes and immune responses by regulating multiple pathways, including ion transport, carbohydrate metabolism, redox reactions, signal transduction, and lipid metabolism, thereby effectively responding to viral infection.

### 3.7 KEGG enrichment analysis of the host DEGs

**Figure 5:**
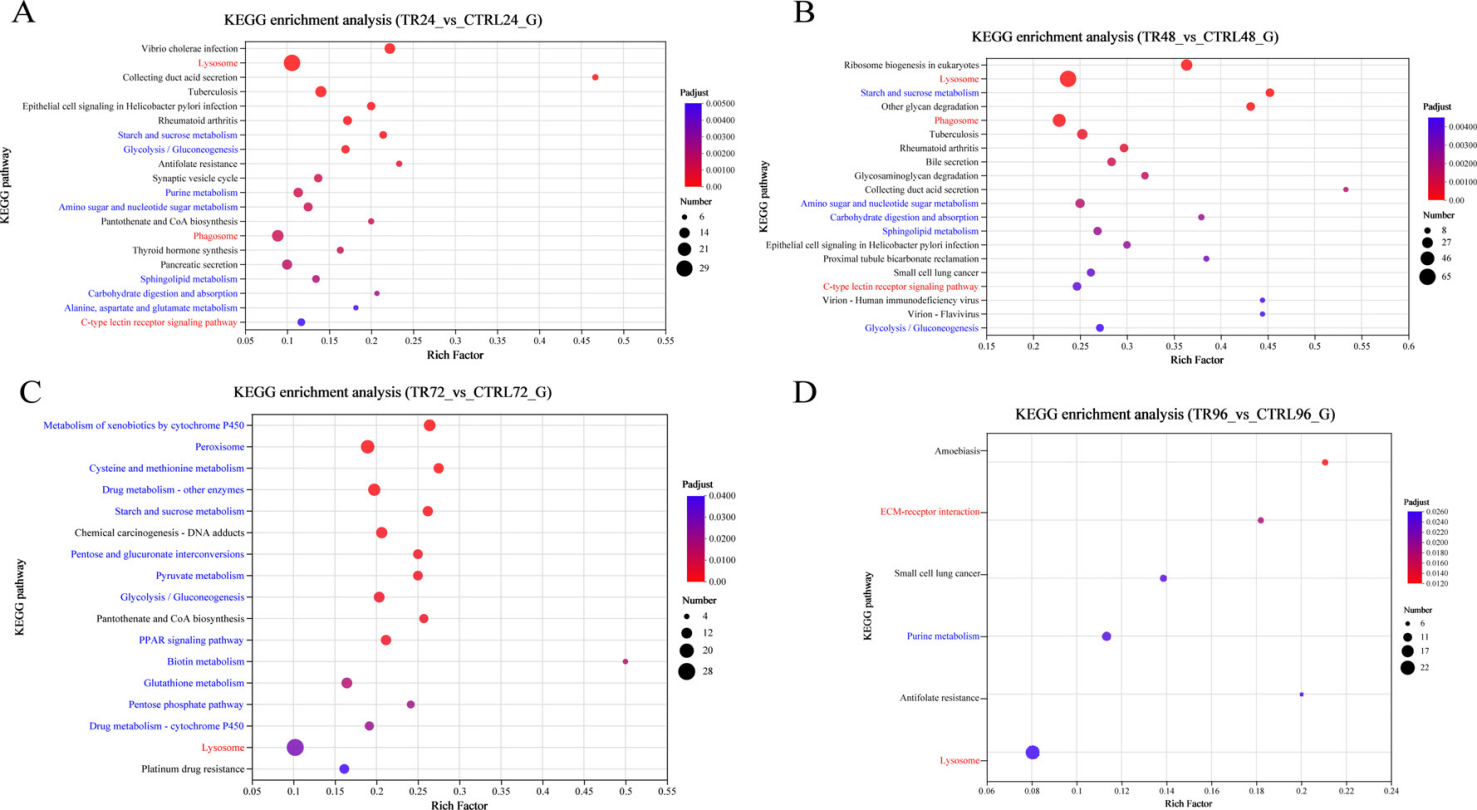
KEGG enrichment analysis bar plots of DEGs. **(A-D)** show the enrichment of all DEGs between the DIV1-infected and control groups at 24, 48, 72, and 96 hpi, respectively. The analysis was performed using Fisher’s exact test, with a significance threshold set to padjust < 0.05. Each plot displays the top 20 KEGG pathway based on the padjust values. The red font represents immune-related pathways, while the blue font showcases pathways related to metabolism, antioxidation, and glycolysis. The y-axis represents the pathway names, while the x-axis shows the rich factor, which is the ratio of the number of enriched genes in the pathway (sample number) to the total number of annotated genes (background number). A larger rich factor indicates a greater degree of enrichment. The size of the dots represents the number of genes in the pathway, and the color of the dots corresponds to different ranges of padjust values.

KEGG pathway enrichment analysis was conducted on the DEGs using an R script, employing Fisher’s exact test as the statistical method. A pathway was considered significantly enriched if the adjusted P value (Padjust) was < 0.05. **Figure 5** presents the top 20 enriched pathways associated with DIV1 infection at 24, 48, 72, and 96 hpi, ordered in ascending sequence according to their adjusted *p*-values. At both 24 and 48 hpi, three immune-related pathways (red font) were identified: Lysosome, Phagosome, and C-type lectin receptor signaling pathway. At 72 hpi, Lysosome showed significant enrichment. At 96 hpi, two immune-related pathways, ECM-receptor interaction and Lysosome, were significantly enriched. DEGs enriched in the Lysosome, Phagosome, and C-type lectin receptor signaling pathway are listed in **Supplementary Table S4**.

In addition, the DEGs are significantly enriched in pathways related to metabolism, antioxidation, and glycolysis (blue font). At 24 hpi, significant enrichment was observed in the following pathways: Starch and sucrose metabolism, Glycolysis/Gluconeogenesis, Purine metabolism, Amino sugar and nucleotide sugar metabolism, Alanine, aspartate and glutamate metabolism, Carbohydrate digestion and absorption, and Sphingolipid metabolism. At 48 hpi, Starch and sucrose metabolism, Amino sugar and nucleotide sugar metabolism, Carbohydrate digestion and absorption, Sphingolipid metabolism, and Glycolysis/Gluconeogenesis continued to be significantly enriched. At 72 hpi, 1105 DEGs were mapped to multiple metabolism-related pathways, including Metabolism of xenobiotics by cytochrome P450, Peroxisome, Cysteine and methionine metabolism, Drug metabolism - other enzymes, Starch and sucrose metabolism, Pentose and glucuronate interconversions, Pyruvate metabolism, Glycolysis/Gluconeogenesis, PPAR signaling pathway, Biotin metabolism, Glutathione metabolism, Pentose phosphate pathway, and Drug metabolism - cytochrome P450. Notably, Peroxisome, Cysteine and methionine metabolism, Glutathione metabolism, and the Pentose phosphate pathway are closely related to antioxidation processes. These pathways help protect cells from oxidative damage by scavenging free radicals and peroxides, facilitating the production of key antioxidants such as glutathione and NADPH. At 96 hpi, Purine metabolism was also significantly enriched. Additionally, significant enrichment of Glycolysis/Gluconeogenesis was observed at 24, 48, and 72 hpi.

### 3.8 Weighted gene co-expression network analysis

**Figure 6:**
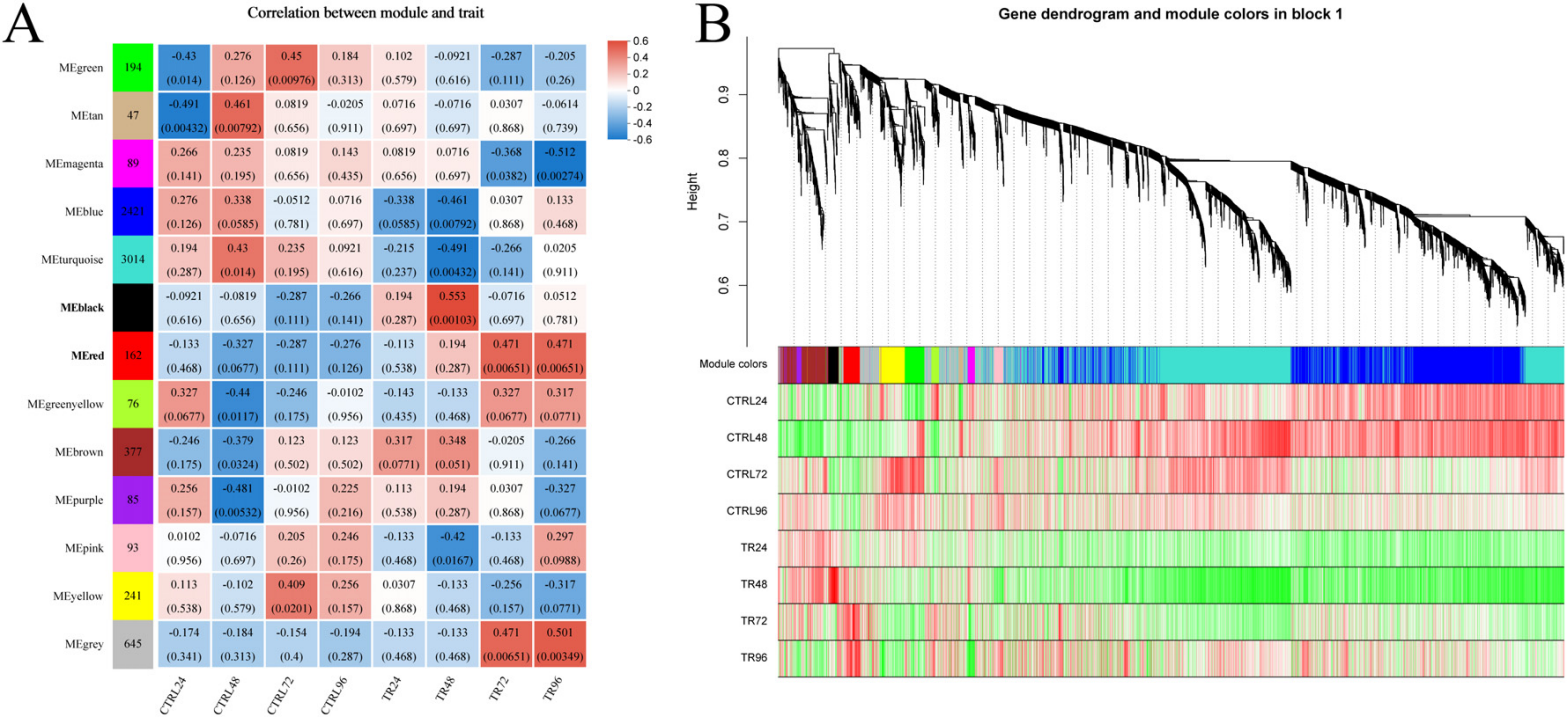
Weighted gene co-expression network analysis. **(A)** Heatmap of module-infection time correlation: The x-axis represents different groups, while the y-axis corresponds to various modules. The leftmost column displays the number of genes within each module. Gray represents genes in this module that have not been assigned to a specific module. On the right side of each group, the correlation coefficient between the module and the phenotype is shown, along with the significance *p*-value (in parentheses). Larger absolute values indicate stronger correlations. Blue denotes a negative correlation, and red represents a positive correlation. **(B)** Gene-phenotype clustering analysis. This analysis visualizes the correlation between genes within the module and phenotypes. The top part of the image shows a hierarchical clustering tree of genes, the middle section indicates the module to which each gene belongs, and the bottom displays a heatmap of gene-phenotype correlations within the module. Each row represents a group, and each column corresponds to a gene within the module. The color intensity reflects the strength of the correlation, with red indicating a positive correlation and green indicating a negative one.

In this study, we performed gene co-expression network analysis based on the 7,544 genes retained after filtering, and finally identified the 13 modules with different expression patterns. These modules were subsequently associated with infection time, and a heat map of module-infection time correlation was generated based on the Spearman correlation coefficient (**Figure 6**). Among these modules, the black module, comprising 100 genes, exhibited a strong positive correlation with the 48-hour post-infection (hpi) time point (r = 0.553, *p* = 0.001), while the red module, consisting of 162 genes, showed a strong positive correlation with both the 72 hpi (r = 0.471, *p* = 0.0065) and 96 hpi (r = 0.471, *p* = 0.0065) time points. Notably, both of these modules were significantly positively correlated with the DIV1-infected group and negatively correlated with the control group at the corresponding time points. **Figure 6B** shows the correlation between genes within the modules and the phenotype. As a result, these two modules were selected for further enrichment analyses due to their potential biological relevance in the context of DIV1 infection.

### 3.9 Characterization of the hub genes

**Figure 7:**
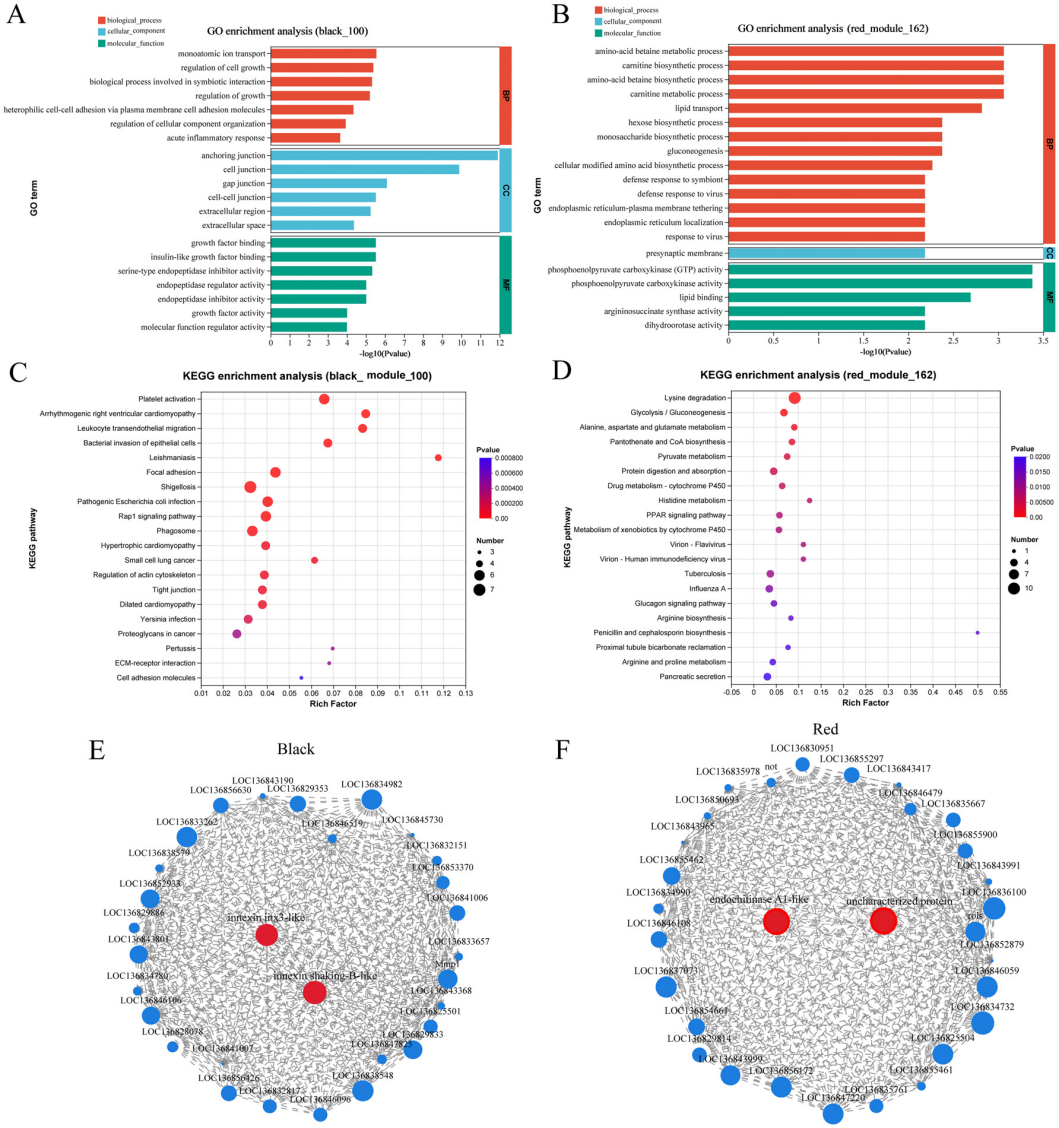
GO enrichment analysis bar plots. **(A, B)** display the GO enrichment analysis of genes in the black and red modules (*p*-value ≤ 0.05, top 20 pathways). **(C, D)** illustrate the KEGG enrichment analysis of genes in the black and red modules (*p*-value ≤ 0.05, top 20 pathways). **(E)** Visualization of the top 30 highly connected nodes in the black module: Each node represents a gene, with nodes displaying greater connectivity considered more significant. **(F)** Visualization of the top 30 highly connected nodes in the red module. Similar to panel **(E)**, this visualization shows the connectivity of the top 30 nodes in the black module.

GO and KEGG enrichment analysis were performed using gene sets from the dark and red modules. The GO enrichment analysis of DEGs in the black module identified that the two most significantly enriched GO terms were anchoring junction and cell junction (**Figure 7A**). Similarly, the GO enrichment analysis of DEGs in the red module highlighted phosphoenolpyruvate carboxykinase (GTP) activity as the most significantly enriched GO term (**Figure 7B**). DEGs in the black and red modules are enriched in 44 and 35 pathways, respectively. Among the top 20 significantly enriched pathways in the black module, immune-related pathways such as platelet activation, leukocyte transendothelial migration, phagosome formation, and ECM-receptor interaction were identified. In contrast, the top five significantly enriched pathways in the red module were all metabolism-related, with the Lysine degradation pathway being the most highly enriched (**Figures 7C, D**). Hub genes were identified based on node connectivity within the target black and red modules. The top 30 genes with the high connectivity were selected from each module, as shown in **Table 5**, and two gene network diagrams were constructed (**Figures 7E, F**). In the black module, the core genes are mainly involved in intercellular communication, including innexin shaking-B-like and innexin inx3-like. Innexins are gap junction proteins, which belong to the transmembrane protein family and are typically associated with gap junctions (31). They play a key role in direct communication between cells. Additionally, leukocyte elastase inhibitors primarily regulate immune responses by inhibiting the activity of elastase, preventing the degradation of elastin. Elastase is usually secreted by immune cells like neutrophils and is involved in inflammatory responses (32). Counteracting excessive elastase activity produced by immune cells helps control inflammation and tissue damage (32). Integrins are receptors on the cell surface that mainly participate in interactions between cells and the extracellular matrix, regulating cell migration, adhesion, and signal transduction (33). In the red module, the hub genes identified include endochitinase A1-like, macrophage mannose receptor 1-like, and caspase-1. These genes play significant roles in various biological processes. Endochitinase A1-like is involved in the degradation of chitin and may contribute to the molting process or the immune response against chitin-containing pathogens in organisms like shrimp. Macrophage mannose receptor 1-like is crucial for the innate immune system, as it recognizes and binds carbohydrate structures on the surface of pathogens, facilitating pathogen recognition and phagocytosis (34). Caspase-1 is a critical protease in regulating inflammation and cell death, primarily through the activation of inflammasomes and the subsequent processing of pro-inflammatory cytokines, thus playing a central role in the immune response and inflammation (35). In summary, the hub genes identified in the black and red modules play crucial roles in intercellular communication, immune responses, and inflammatory regulation.

**Table 5:** Characterization of key genes in the black and red modules.

Gene name	Gene Description
Black module	
LOC136846104	innexin shaking-B-like
LOC136846105	innexin inx3-like
LOC136838548	platelet-derived growth factor subunit B-like, transcript variant X1
LOC136834982	uncharacterized protein
LOC136833262	uncharacterized peptidase C1-like protein F26E4.3
Mmp1	Matrix metalloproteinase 1, transcript variant X1
LOC136852933	uncharacterized lncRNA
LOC136829833	ipis-1-like, transcript variant X1
LOC136846106	innexin inx3-like, transcript variant X1
LOC136843801	phenoloxidase-activating enzyme-like
LOC136829353	X-linked interleukin-1 receptor accessory protein-like 2, transcript variant X2
LOC136856426	uncharacterized lncRNA
LOC136841006	uncharacterized protein, transcript variant X2
LOC136856630	uncharacterized protein, transcript variant X3
LOC136825501	uncharacterized protein
LOC136846096	innexin inx2-like
LOC136832817	triosephosphate isomerase-like
LOC136853370	uncharacterized protein, transcript variant X1
LOC136828078	uncharacterized lncRNA
LOC136829886	leukocyte elastase inhibitor-like, transcript variant X1
LOC136847825	uncharacterized lncRNA
LOC136832151	uncharacterized protein
LOC136846519	integrin beta pat-3-like
LOC136834780	uncharacterized protein, transcript variant X1
LOC136833657	leukocyte elastase inhibitor-like, transcript variant X2
LOC136838579	integrin beta-PS-like, transcript variant X1
LOC136843368	transient receptor potential channel pyrexia-like
LOC136843190	prostaglandin G/H synthase 2-like
LOC136845730	integrin beta-PS-like, transcript variant X1
LOC136841007	uncharacterized protein
Red module	
LOC136843580	uncharacterized protein
LOC136846058	endochitinase A1-like
LOC136834732	rhomboid-related protein 1-like, transcript variant X9
LOC136836100	macrophage mannose receptor 1-like, transcript variant X1
LOC136837073	uncharacterized protein
LOC136846059	uncharacterized protein
LOC136856172	receptor-type tyrosine-protein phosphatase F-like
LOC136847220	protein split ends-like, transcript variant X1
LOC136825504	macrophage mannose receptor 1-like
rols	rolling pebbles, transcript variant X1
LOC136843999	–
LOC136855462	caspase-1-B-like, transcript variant X3
LOC136854661	uncharacterized protein, transcript variant X1
LOC136846108	innexin inx2-like
LOC136855297	uncharacterized protein
LOC136855900	(2S)-3-sulfopropanediol dehydratase-like
LOC136835667	spectrin alpha chain, non-erythrocytic 1-like, transcript variant X1
LOC136829814	carbohydrate sulfotransferase 5-like
LOC136830951	uncharacterized protein
LOC136835761	alpha-actinin-1-like
LOC136846479	uncharacterized protein, transcript variant X6
LOC136834990	NFX1-type zinc finger-containing protein 1-like, transcript variant X1
not	non-stop, transcript variant X1
LOC136855461	uncharacterized protein
LOC136835978	–
LOC136850693	extended synaptotagmin-2-like, transcript variant X1
LOC136843991	–
LOC136843417	uncharacterized protein, transcript variant X1
LOC136843965	gamma-butyrobetaine dioxygenase-like
LOC136852879	uncharacterized protein, transcript variant X2

“-” represents no gene function description.

### 3.10 Validation of RNA-seq results by qRT-PCR

**Figure 8:**
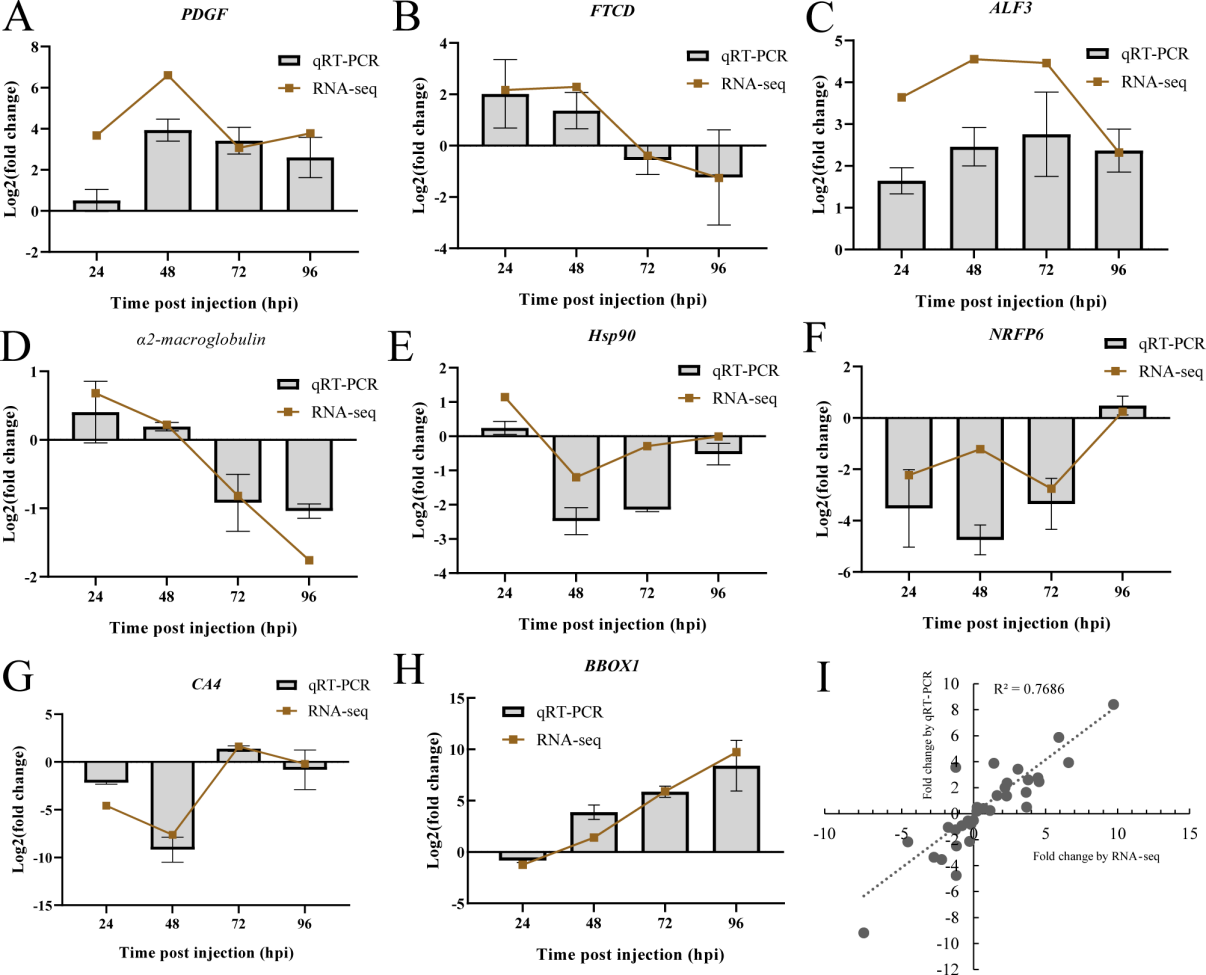
Validation of RNA-seq results using qRT-PCR. **(A–H)** Comparison of 8 DEGs expression profiles as analyzed by Illumina sequencing and qRT-PCR. The x-axis represents the time post-injection, and the y-axis displays the log2(fold change). **(I)** The qRT-PCR results were compared with the RNA-seq quantification data, and the Pearson correlation coefficient (R²) was calculated to assess the positive linear correlation between the two methods.

To validate the mRNA expression profiles based on Illumina sequencing results, a total of 8 genes were selected for qRT-PCR. As illustrated in **Figure 8**, the expression patterns of the tested genes were nearly consistent between the two methods. The correlation analysis showed a strong linear correlation (Pearson correlation coefficient, R^2^ = 0.77) between gene transcript levels, confirming the reliability of the RNA-seq data and supporting the observed changes in gene expression in response to DIV1 infection.

## 4 Discussion

*M. rosenbergii* is a valuable crustacean known for its vibrant coloration, excellent flavor, and high economic worth. However, the widespread occurrence of DIV1 has severely threatened the sustainability of its aquaculture industry. Gaining a thorough understanding of the immune response of *M. rosenbergii* to DIV1 infection could offer novel strategies for disease control and management. In this study, we used RNA-seq to investigate the dynamic immune response in the hepatopancreas of *M. rosenbergii* at different time points post-infection. Notably, this study represents the first in-depth investigation into the transcriptomic alterations triggered by DIV1 infection in *M. rosenbergii*, providing valuable insights into the molecular mechanisms underlying host-pathogen interactions.

In this study, a large number of DIV1 gene sequences were detected in our transcriptomic data, although the functions of most of these viral genes remain unclear. Notably, the genes encoding the hypothetical protein KM509 gp050 and Ca²^+^-binding RTX toxin-related protein exhibited the highest abundance at 24 hpi, suggesting that these two genes may play crucial roles in the early stages of DIV1 infection. As the infection progressed, additional transcripts were identified at 48 hpi, including those associated with the DNA-dependent RNA polymerase II largest subunit, ribonuclease III, 116L, hypothetical protein KM509 gp059, and head decoration. Notably, the DNA-dependent RNA polymerase II largest subunit is an essential component of the host cell transcriptional machinery, functioning as the core subunit of RNA polymerase II, which is responsible for the synthesis of mRNA. The expression of this gene may indicate that DIV1 relies on the host cell transcription system to replicate and express viral genes. Ribonuclease III, typically involved in RNA degradation and processing, may play a role in regulating the stability, processing, or cleavage of viral RNA during infection, thereby influencing the viral life cycle and modulating the host cell’s response mechanisms (36). The head decoration-related protein may be a virus-specific structural or accessory protein involved in virus assembly, surface modification, or facilitating viral propagation within host cells (37). At 72 hpi and 96 hpi, a significantly higher abundance of the 068R transcript was detected, indicating that this gene plays a critical role in the later stages of DIV1 infection, warranting further investigation.

At 24 hpi, the pathways most significantly influenced by DEGs primarily include ATPase-coupled monoatomic cation transmembrane transporter activity, catalytic activity, ATPase activity, coupled to transmembrane movement of ions, rotational mechanism, proton-transporting ATPase activity, rotational mechanism, ATPase-coupled ion transmembrane transporter activity, and ATPase-coupled transmembrane transporter activity. The enrichment of these GO terms suggests that DIV1 infection may support viral replication and persistent infection by affecting the host cell’s ATPase-related activities, particularly processes related to ion transport and energy metabolism. Further investigation into the specific role of ATPase-related activities in DIV1 infection could provide potential targets for the development of new antiviral therapeutic strategies. Compared to other infection time points, samples collected at 48 h exhibited the most DEGs, indicating that large-scale transcriptional alterations of the host genes occurred. At this time, the enrichment of GO terms related to the Nucleolus, Hydrolase activity, rRNA metabolic process, rRNA processing, and ribosome biogenesis reflect the host’s metabolic adjustments to viral infection. The nucleolus is responsible for the synthesis and processing of ribosomal RNA, as well as the maturation of ribosomes (38). The enrichment of the Nucleolus may be associated with increased protein synthesis. Hydrolases can degrade proteins within the cell, eliminating damaged or unnecessary proteins, thereby helping the cell maintain normal function. This process is crucial for responding to cellular stress induced by viral infections. Activation of the Nucleolus and Hydrolase activity may be one of the host cell’s strategies to combat viral invasion. Stress factors such as hypoxia and microbial infections can lead to oxygen depletion and elevated levels of reactive oxygen species (ROS), triggering oxidative stress in cells (39). As the infection progresses, the significant enrichment of oxidoreductase activity suggests that the host may regulate redox states to clear ROS generated by the infection and protect cells from oxidative damage. Furthermore, the enhancement of carbohydrate-binding function may suggest that the host regulates viral entry, immune response, or other cellular reactions through interactions with carbohydrate molecules. This stage could be a critical point for the host to mount an immune response against viral infection. In summary, DIV1 infection significantly impacts the host’s core physiological functions, including energy metabolism, ion homeostasis, carbohydrate metabolism, and redox balance. The virus may alter these critical processes to suppress the host’s antiviral capacity while utilizing host resources to support its own replication and spread. Analyzing these enriched GO terms provides avenues for further exploration: Investigating enriched immune-related molecular pathways (e.g., oxidoreductase activity and carbohydrate metabolism) as potential antiviral targets and how the virus exploits host ATPase activity and carbohydrate binding to uncover its infection strategies.

KEGG enrichment analysis revealed significant changes in immune-related pathways during DIV1 infection. At 24 and 48 hpi, the Lysosome, Phagosome, and C-type lectin receptor signaling pathways were significantly enriched. The Lysosome pathway continued to be significantly enriched at 72 hpi, and by 96 hpi, both the ECM-receptor interaction and Lysosome pathways were significantly enriched. Among the pathways mentioned above, the Lysosome and Phagosome pathways are the most impacted. The Lysosome pathway remained consistently enriched throughout the infection cycle, emphasizing its essential role in viral clearance, autophagy, and host defense. Previous studies have highlighted the presence of lysosomes in the granulosa hemocytes of various crustacean species (40). Phagocytosis or receptor-mediated endocytosis plays a crucial role in immune defense, where phagocytic cells capture pathogens or antigens, enclosing them in phagosomes (41). After phagocytosis, lysosomes fuse with these phagosomes and endosomes, forming mature phagolysosomes, which are crucial for the degradation and elimination of internal pathogens (42). Next, we will discuss the genes of interest from Lysosome, Phagosome, and C-type lectin receptor signaling pathways. Cathepsin D (43), Procathepsin L-like (44), Beta-hexosaminidase subunit alpha-like (45) primarily function in lysosomes, participating in the degradation of proteins, lipids, and carbohydrates. Their upregulation may indicate enhanced autophagy or lysosome-related degradation under stress conditions. Cathepsin B (CTSB) is a cysteine protease and a member of the lysosomal cathepsin family, playing a key role in processes such as cell autophagy and apoptosis, which are two forms of programmed cell death (46). It has been linked to cancer progression and plays a role in antigen processing by antigen-presenting cells (47). Previous research in *Fenneropenaeus chinensis* showed that *cathepsin B* expression increases in the gill, hepatopancreas, and muscle after WSSV infection, suggesting its role in virus resistance (48). A recent study also reported the up-regulation of *catB* in hemocytes and the hepatopancreas in response to VpAHPND infection (49). However, in contrast to these findings, our study shows that both *cathepsin B* and *cathepsin L-like* are downregulated at 48 hpi. The observed downregulation of *cathepsin B* and *cathepsin L-like* suggests that the virus may manipulate host cell pathways by inhibiting cell death and autophagy, thereby prolonging the survival of infected cells and facilitating the persistence of viral infection. The underlying reasons for these differences require further investigation. The V-type proton ATPase (V-ATPase) is an ATP-dependent pump crucial for maintaining acidic environments in intracellular organelles, such as secretory granules, endosomes, and lysosomes, as well as in extracellular spaces. This enzyme complex is crucial for various physiological processes, including membrane trafficking, protein degradation, pH homeostasis, and the regulation of intracellular signaling. Furthermore, V-ATPase is implicated in several pathological conditions, such as viral and toxin entry, drug resistance, and the survival, migration, and invasion of cancer cells (50). The downregulation of V-ATPase components may indicate compromised lysosomal function. Lysosomes are essential for cellular clearance, responsible for degrading viruses, damaged organelles, and metabolic waste. A reduction in lysosomal acidification could impair the host cell’s ability to effectively eliminate viruses and respond to infection. This could represent a viral strategy to evade the host immune defense by disrupting lysosomal function, thus promoting viral survival and dissemination. The downregulation of these genes may reflect adaptive changes in host cells that facilitate a more favorable environment for viral replication. Viral infections typically induce alterations in the host cell’s physiological state and functional pathways to promote viral propagation, with gene downregulation potentially serving as a key element of this adaptive response. Macrophage mannose receptor 1-like, C-type lectin domain family 17 members, and CD209 antigen-like belong to C-type lectin receptors, which recognize pathogens through sugar structures, mediating the activation of immune cells and the clearance of pathogens. They play a primary role in immune responses, particularly in the early stages of pathogen infection, by recognizing and binding to specific sugar molecules on pathogens, initiating the host’s immune defense mechanism. Perlucin-like protein (PLP) is another typical C-type lectin. It plays a crucial role in *L. vannamei*’s immune response by binding and agglutinating bacteria, influencing phagocytosis and AMP expression (51), and activating immune-related and apoptosis genes during *V. parahaemolyticus* infection (52). In this study, we found that DIV1 infection induced a sustained upregulation of the gene encoding Perlucin-like protein, highlighting its important role in the innate immunity of *M. rosenbergii*, warranting further investigation. In conclusion, the sustained activation of the Lysosome, Phagosome, and C-type lectin receptor signaling pathways emphasizes their important roles in the host’s response to viral infection. Future research could focus on these related genes to explore the regulatory mechanisms after the virus infects the shrimp.

Metabolism-related pathways were significantly enriched throughout the infection process; however, the specific metabolic pathways exhibited distinct variations as the infection progressed. At 24 and 48 hpi, Starch and sucrose metabolism, Amino sugar and nucleotide sugar metabolism, Carbohydrate digestion and absorption, Sphingolipid metabolism, and Glycolysis/Gluconeogenesis were all significantly enriched. This suggests that in the early to mid-stage of infection, host cells may activate these key metabolic pathways to meet the energy demands and regulate cellular functions in response to the infection. At 72 hpi, 13 of the top 17 enriched pathways of DEGs were related to metabolism, including those associated with antioxidant defense, such as the Peroxisome, Cysteine and methionine metabolism, and Glutathione metabolism. These pathways help eliminate ROS and reduce oxidative stress damage caused by viral infection. PPARs are key regulators of metabolism, involved in maintaining the balance of glucose and lipid levels (53, 54). Studies have shown that the PPAR signaling pathway enhances the proliferation and suppresses the apoptosis of colon cancer cells (55). The activation of this pathway might meet the needs of DIV1 replication by promoting cell proliferation. Moreover, the Glycolysis/Gluconeogenesis pathway was significantly activated at 24, 48, and 72 hpi, a hallmark feature of the Warburg effect (14, 56). First identified by Warburg in the 1930s, the Warburg effect describes the metabolic reprogramming that occurs in tumors and cancer cells to fulfill their elevated energy requirements and to support the rapid synthesis of macromolecules (57). This effect involves a shift away from aerobic respiration, even in oxygen-rich environments, allowing cancer cells to evade apoptosis and immune detection. At the same time, it enhances glycolysis, providing the energy and metabolites necessary for cancer cell proliferation (58). Furthermore, it has been discovered that certain viruses in vertebrates can trigger this phenomenon, such as the human cytomegalovirus (HCMV) (59) and human papillomavirus (HPV) (60). In a pioneering study on WSSV, Chen et al. investigated the glycolytic alterations induced during non-mammalian viral infections. Their findings revealed increased glucose consumption, elevated plasma lactate concentrations, and enhanced activity of glucose-6-phosphate dehydrogenase (G6PDH), all of which suggest a shift towards glycolysis. Furthermore, they observed no significant change in the ADP/ATP ratio and a lower level of oxidative stress in comparison to uninfected controls at 12 hpi. These metabolic changes closely resemble the Warburg effect, a phenomenon typically associated with mammalian cells during viral infections or tumorigenesis (58). Similar result was reported in another article (61). A separate study on DIV1 infection in *Marsupenaeus japonicus* identified significant enrichment in three hallmark pathways of the Warburg effect, including pyruvate metabolism, glycolysis/gluconeogenesis, and the TCA cycle, as well as the triose-phosphate isomerase activity GO terms (62). Similarly, a study on *Litopenaeus vannamei* infected with DIV1 demonstrated a significant upregulation of all glycolytic genes, accompanied by a marked increase in pyruvate and lactate levels throughout the infection process (63). Furthermore, DIV1 infection also induced metabolic dysregulation and triggered the Warburg effect in other species, including *Penaeus monodon* (14), *Fenneropenaeus merguiensis* (15), and *Cherax quadricarinatus* (64). Our results further support previous studies. Collectively, these studies emphasize the heightened energy demands during pathogen invasion, suggesting that the organism may modulate hepatopancreatic metabolism to mitigate the metabolic disturbances induced by the infection. In conclusion, this study reveals the dynamic changes in host immune, metabolic, and antioxidant pathways during DIV1 infection, providing important insights into the molecular mechanisms of DIV1 pathogenesis. Future research should integrate transcriptomic, proteomic, and metabolomic data to validate the functions of key pathways and explore potential antiviral intervention strategies.

The DEGs in the red module were mapped to the GO terms associated with Phosphoenolpyruvate carboxykinase (GTP) activity. Phosphoenolpyruvate carboxykinase is a key metabolic enzyme that plays a crucial role in regulating gluconeogenesis (65), which suggests that the virus may manipulate the host’s metabolic pathways to optimize its energy supply, thereby facilitating viral proliferation. We further discovered three hub genes, primarily involved in cell-cell communication (innexin shaking-B-like and innexin inx3-like) and endochitinase A1-like. Researchers originally identified innexin as the structural protein of gap junctions in *Drosophila* and *Caenorhabditis elegans* (66, 67). In this study, the expression of innexin shaking-B-like and innexin inx3-like was significantly upregulated following DIV1 infection, and their connection values were high within the co-expression module, highlighting their potential importance in immune regulation in *M. rosenbergii*. Chitin is predominantly present in the exoskeletons of arthropods (such as insects and crustaceans), as well as in the cell walls of fungi and algae (68). Chitinase can hydrolyze chitin, inhibiting the growth of fungi (69). Although the virus itself does not contain chitin, host cells may upregulate endochitinase A1-like during infection as a nonspecific immune response to counter potential secondary infections (such as bacterial or fungal infections), which can exacerbate the clinical manifestations of the viral infection. In this context, it was observed that the gene encoding endochitinase A1-like was upregulated after DIV1 infection, suggesting that this gene may play a crucial role in the invasion and replication of DIV1. Our findings align with previous studies, where exposure of *Penaeus monodon* to *Streptococcus agalactiae* and *Vibrio harveyi* resulted in a significant upregulation of *chitinase-5* in the hepatopancreas and gills, providing further evidence for the involvement of chitinase in immune defense mechanisms (70).

Furthermore, a total of 15 DEGs (14 upregulated and 1 downregulated) shared across four time points post-infection attracted our attention, as they may play a crucial role in the host’s response to DIV1 infection. Proteases secreted by pathogenic organisms are pivotal in facilitating host invasion, pathogen dissemination, immune evasion, and the disruption of essential host functions (71). Consequently, hosts have evolved protease inhibitors as part of their defense mechanism to neutralize proteases and reduce the risk of illness and death. These protease inhibitors can be broadly categorized into two classes: (i) active site inhibitors, which directly bind to the active site of the protease, and (ii) alpha-2-macroglobulins (α2M), which inhibit protease activity through a conformational change, effectively trapping the protease within the α2M molecule in an irreversible manner, a process known as the physical entrapment mechanism (72). Invertebrates employ α2M, a highly abundant plasma protein, as a broad-spectrum protease inhibitor, playing pivotal roles in a wide range of immune responses across diverse species (73). For example, it is involved in processes like phagocytosis in *Ixodes ricinus* (74) and *Anopheles gambiae* (75), and blood clotting in both *Pacifastacus leniusculus* (76) and *Limulus polyphemus* (77). Notably, in *Vibrio harveyi*-infected *Penaeus monodon*, Pmα2M was found to be the most significantly altered protein in both hemocytes (78) and the lymphoid organ (79), highlighting its important role in immune responses against pathogens. In this study, the sustained upregulation of α2M suggests that the host is facing immune stress, attempting to protect itself from the pathogen by regulating immune responses and inhibiting inflammation and protease activity. Serum amyloid A (SAA) is an acute-phase protein that is significantly elevated—up to 1000-fold—during infection and inflammation, and is widely recognized as a key marker of inflammation (80). In addition to its role as an inflammation marker, SAA may function in a cytokine-like manner, initiating signaling through toll-like receptors (TLRs) to further amplify the inflammatory response (80). It plays a pivotal role in the immune response, with its elevated expression also being observed in *Marsupenaeus japonicus* (Japanese tiger prawn) infected with WSSV (22), thereby underscoring its involvement in crustacean immune defense. Furthermore, SAA has been proposed as part of a biomarker panel for identifying physiological changes indicative of tumor progression and the host’s immune response (81). In the present study, the genes LOC136840094 and LOC136840095, which encode serum amyloid A-5 protein-like (SSA5), were consistently upregulated, further emphasizing the critical role of SAA in immune modulation. The prophenoloxidase (proPO) system is a critical component of the innate immune response in many invertebrates (82). Phenoloxidase (PO), which is involved in melanin production, is initially synthesized as an inactive precursor known as prophenoloxidase (proPO). Upon detection of pathogens, a serine protease cascade is triggered, activating proPO by converting it into its active form, PO (83). This activation is facilitated by prophenoloxidase activating enzyme (PPAE), which cleaves proPO to produce active PO (84). The end product of the proPO cascade, melanin, is not only involved in pigmentation but also exhibits protective functions, including fungistatic, bacteriostatic, and antiviral properties (82). This mechanism provides invertebrates with a robust defense system against various pathogens, contributing significantly to their innate immune responses. In our study, the sustained up-regulated expression of LOC136843801 (phenoloxidase-activating enzyme-like) re-emphasized the immune defense role of phenoloxidase. Platelet-derived growth factor (PDGF) is a major mitogen for connective tissue cells, such as fibroblasts and smooth muscle cells (85). As a potent mitogen and chemoattractant, PDGF has been shown to disrupt vascular homeostasis by inducing inflammation, oxidative stress, and phenotype transition (86). The enhanced signaling of PDGF and its receptor (PDGFR) has become a hallmark of various diseases, including cancer and atherosclerosis (87). Therefore, inhibiting PDGF-PDGFR signaling has become an important area of research in cancer therapy (87). Furthermore, the combined inhibition of PDGF and vascular endothelial growth factor (VEGF) is considered a promising strategy for suppressing angiogenesis in tumor progression (88). In this study, the sustained upregulation of the gene encoding platelet-derived growth factor subunit B-like may have contributed to the progression of the disease caused by DIV1, and further research will focus on this gene. Alpha-amylase 1-like, putative aldolase class 2 protein PA3430, and pantetheinase-like may play important roles in cellular energy metabolism and metabolite conversion. Specifically, the gene encoding rhodanese domain-containing protein CG4456-like showed sustained downregulation. Studies suggest that the rhodanese domain is typically associated with sulfur transferase or phosphatase activity (89), and its precise biological function requires further investigation. In conclusion, these genes provide potential clues for investigating the innate immunity and disease defense mechanisms of *M. rosenbergii* in response to DIV1 infection.

In conclusion, our study is the first to attempt a transcriptomic time-course analysis of the response of *M. rosenbergii* to DIV1. We identified a total of 3,339 DEGs, with Lysosome, Phagosome, and C-type lectin receptor signaling pathways, as well as ECM-receptor interactions, being the most significantly affected immune-related pathways. Several metabolism-related pathways, such as Starch and sucrose metabolism, Glycolysis/Gluconeogenesis, Purine metabolism, Metabolism of xenobiotics by cytochrome P450, and Peroxisome, were significantly altered during DIV1 infection. Based on WGCNA, we identified two hub modules closely related to infection time, characterized their biological functions, highlighted the involved signaling pathways, and pinpointed a series of hub genes. These findings contribute to a deeper understanding of the immune response to DIV1 in *M. rosenbergii*. Consequently, our results lay the foundation for further investigation into the molecular mechanisms underlying the immune response of *M. rosenbergii* to DIV1.

## Data Availability

The datasets presented in this study can be found in online repositories. The names of the repository/repositories and accession number(s) can be found in the article/**Supplementary Material**.
