# Supplementary material for: Temporal changes in the transcriptome profile of Macrobrachium rosenbergii in response to decapod iridescent virus 1 infection
Source: Front Immunol. 2025 Apr 10;16:1575476. doi: 10.3389/fimmu.2025.1575476 (PMC12018387; doi:10.3389/fimmu.2025.1575476)
Supplement: Supplementary file 1 [file DataSheet1.zip › Supplementary Figures and Tables.DOCX]

**Figure S1**


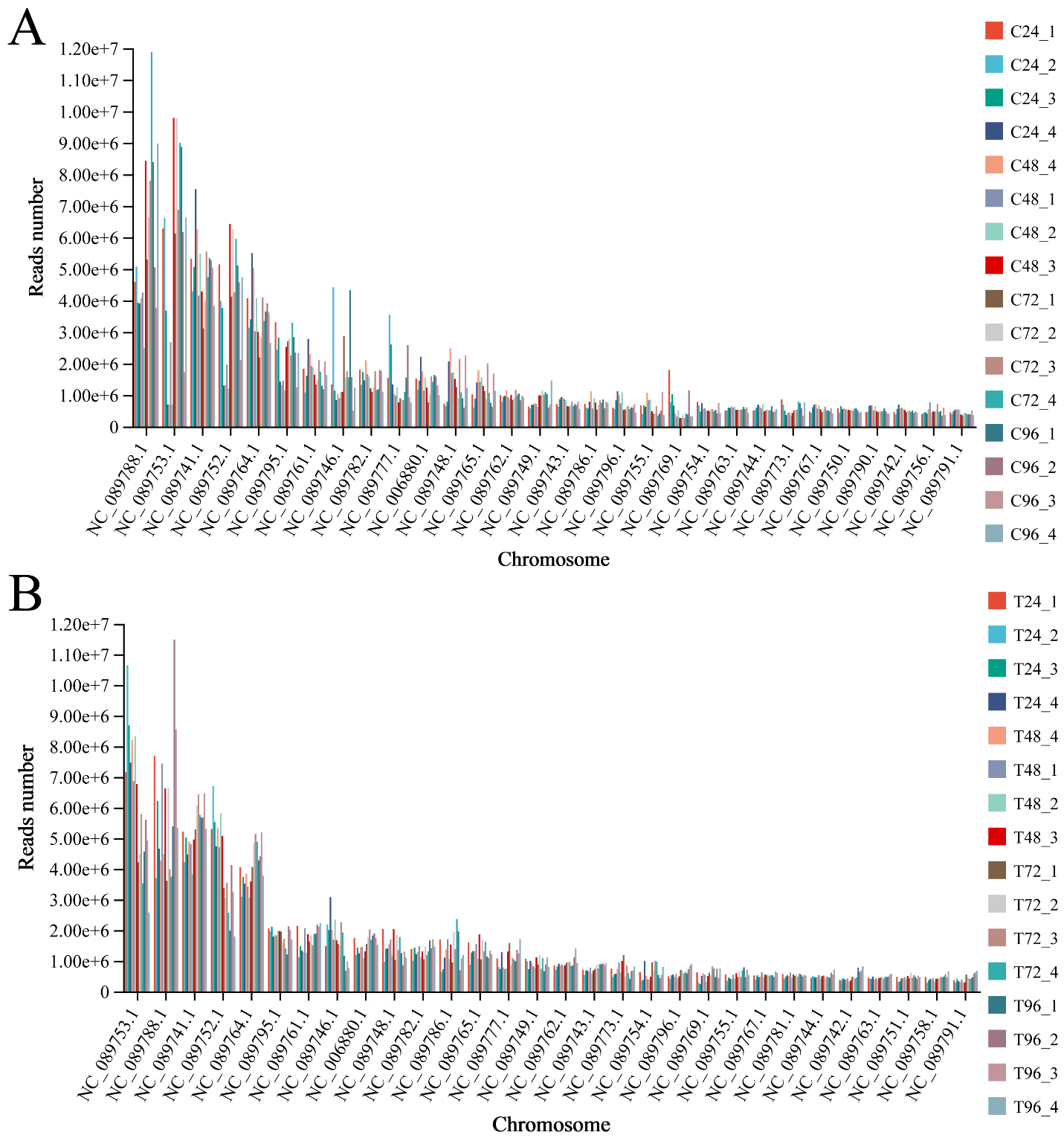


**Figure S1**. Reads distribution in chromosomes. **(A)** and **(B)** represent the distribution of mapped reads on chromosomes in the control group and the DIV1-infected group, respectively. The x-axis represents the distribution of reads across the top 30 chromosomes, while the y-axis indicates the number of mapped reads located on each chromosome.

**Figure S2**


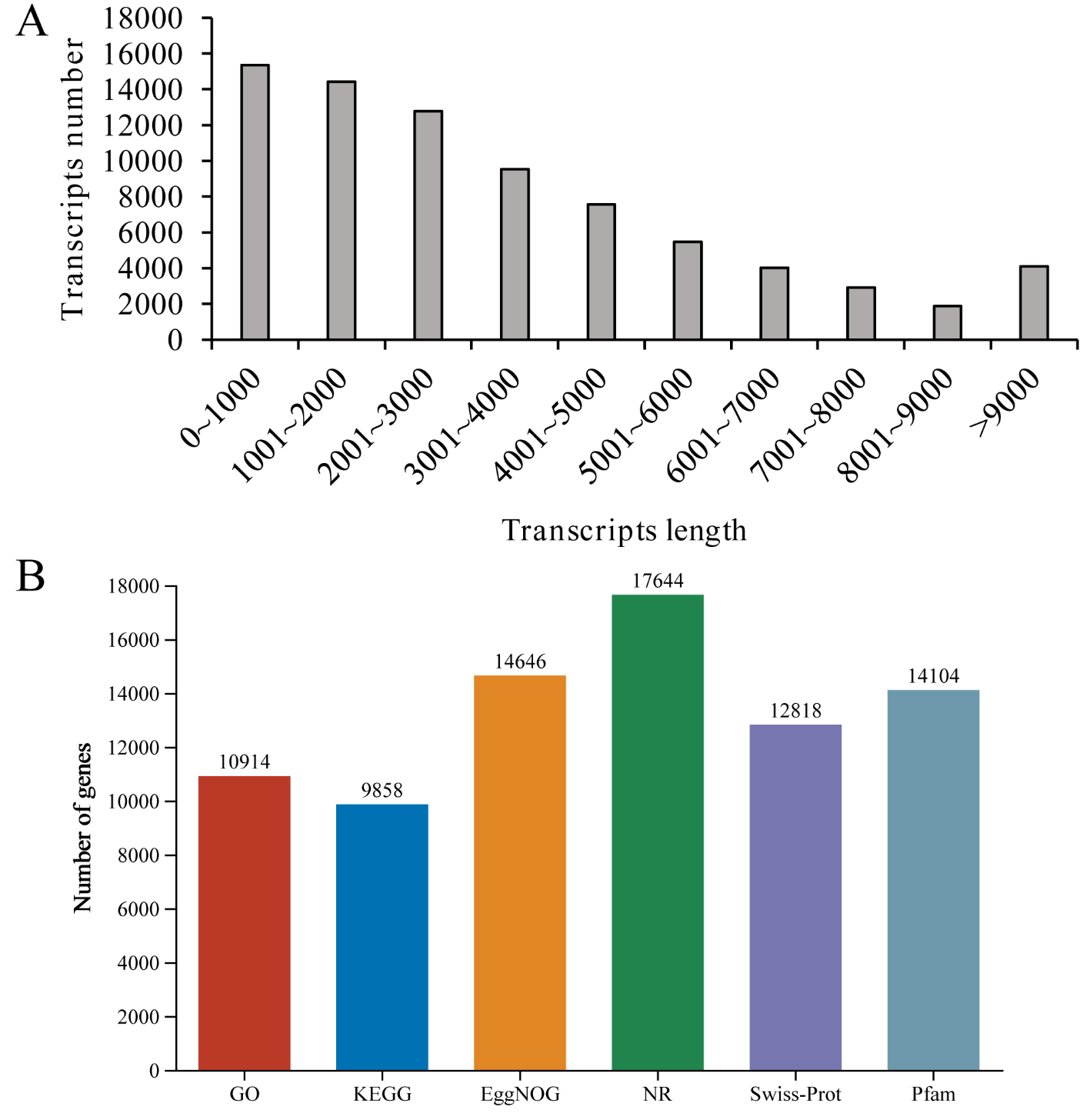


**Figure S2**. Length distribution of transcripts **(A)** and functional annotation of reference genes **(B)**.

Table S1. The distribution of reads across different regions of the reference genome.

| Sample | CDS | Intergenic | Introns | 3’UTR | 5’UTR |
| --- | --- | --- | --- | --- | --- |
| C24_1 | 42396084.0(67.06%) | 1228902.0(1.94%) | 3157932.0(5.0%) | 9809668.0(15.52%) | 6627489.0(10.48%) |
| C24_2 | 37853882.0(66.53%) | 1189179.0(2.09%) | 2655923.0(4.67%) | 9035786.0(15.88%) | 6159387.0(10.83%) |
| C24_3 | 38096435.0(63.93%) | 1637775.0(2.75%) | 2972596.0(4.99%) | 10928905.0(18.34%) | 5957822.0(10.0%) |
| C24_4 | **32422435.0(56.33%)** | 2202787.0(3.83%) | 4511658.0(7.84%) | **11453567.0(19.9%)** | 6963368.0(12.1%) |
| C48_1 | 34647834.0(64.11%) | 1627426.0(3.01%) | 2828591.0(5.23%) | 9568007.0(17.71%) | 5368544.0(9.93%) |
| C48_2 | 30989776.0(58.58%) | **2063427.0(3.9%)** | 3663690.0(6.93%) | 10204859.0(19.29%) | 5977097.0(11.3%) |
| C48_3 | 43954867.0(69.36%) | 1192212.0(1.88%) | **2586152.0(4.08%)** | 9289903.0(14.66%) | 6351823.0(10.02%) |
| C48_4 | 36644541.0(60.68%) | 2052415.0(3.4%) | 4370503.0(7.24%) | 10820381.0(17.92%) | 6499537.0(10.76%) |
| C72_1 | 37105431.0(69.11%) | 1131766.0(2.11%) | 2368947.0(4.41%) | 8416535.0(15.68%) | **4669207.0(8.7%)** |
| C72_2 | 43347478.0(69.26%) | 1204713.0(1.92%) | 2591517.0(4.14%) | 9466762.0(15.13%) | 5976547.0(9.55%) |
| C72_3 | 44850611.0(66.89%) | 1545125.0(2.3%) | 3662569.0(5.46%) | 10241270.0(15.27%) | 6754082.0(10.07%) |
| C72_4 | 45812842.0(69.68%) | 1185799.0(1.8%) | 2874037.0(4.37%) | 9214787.0(14.02%) | 6656227.0(10.12%) |
| C96_1 | 43983085.0(67.72%) | 1390270.0(2.14%) | 3227997.0(4.97%) | 9785517.0(15.07%) | 6561995.0(10.1%) |
| C96_2 | 36196415.0(64.42%) | 1326093.0(2.36%) | 3104014.0(5.52%) | 9282464.0(16.52%) | 6276243.0(11.17%) |
| C96_3 | 35067717.0(65.23%) | 1408490.0(2.62%) | 3185150.0(5.93%) | 8361457.0(15.55%) | 5733198.0(10.67%) |
| C96_4 | 39087875.0(69.87%) | 1010223.0(1.81%) | 2385466.0(4.26%) | 7980267.0(14.27%) | 5476114.0(9.79%) |
| T24_1 | 44616314.0(69.41%) | 1107876.0(1.72%) | 3285474.0(5.11%) | 8669635.0(13.49%) | 6602925.0(10.27%) |
| T24_2 | 37774952.0(68.91%) | 933314.0(1.7%) | 2654848.0(4.84%) | 8069377.0(14.72%) | 5388227.0(9.83%) |
| T24_3 | 39779403.0(67.08%) | 1204410.0(2.03%) | 3108204.0(5.24%) | 9010609.0(15.19%) | 6202936.0(10.46%) |

Table S1. (continued)

| Sample | CDS | Intergenic | Introns | 3’UTR | 5’UTR |
| --- | --- | --- | --- | --- | --- |
| T24_4 | 38778692.0(66.81%) | 1207158.0(2.08%) | 2991316.0(5.15%) | 9478607.0(16.33%) | 5586815.0(9.63%) |
| T48_1 | 43591919.0(69.91%) | 1052332.0(1.69%) | 2748142.0(4.41%) | 8507201.0(13.64%) | 6453453.0(10.35%) |
| T48_2 | 37574168.0(69.91%) | 888101.0(1.65%) | 2525180.0(4.7%) | 7756263.0(14.43%) | 5001673.0(9.31%) |
| T48_3 | **44941956.0(70.84%)** | 1010143.0(1.59%) | 2890547.0(4.56%) | **8142198.0(12.83%)** | 6457813.0(10.18%) |
| T48_4 | 40228825.0(68.98%) | **920180.0(1.58%)** | 3112171.0(5.34%) | 8176193.0(14.02%) | 5882714.0(10.09%) |
| T72_1 | 36265301.0(65.44%) | 1092682.0(1.97%) | 3178361.0(5.74%) | 8889994.0(16.04%) | 5988325.0(10.81%) |
| T72_2 | 40684795.0(66.6%) | 1234091.0(2.02%) | 3785813.0(6.2%) | 8662969.0(14.18%) | 6723738.0(11.01%) |
| T72_3 | 38171276.0(64.78%) | 1169398.0(1.98%) | 3899946.0(6.62%) | 9090786.0(15.43%) | 6593793.0(11.19%) |
| T72_4 | 39056068.0(65.12%) | 1370462.0(2.28%) | **4131356.0(6.89%)** | 9366423.0(15.62%) | 6054529.0(10.09%) |
| T96_1 | 35494097.0(64.38%) | 1226956.0(2.23%) | 3488548.0(6.33%) | 8501694.0(15.42%) | 6420550.0(11.65%) |
| T96_2 | 40736169.0(66.23%) | 1330592.0(2.16%) | 3381067.0(5.5%) | 8666930.0(14.09%) | 7388292.0(12.01%) |
| T96_3 | 42211066.0(64.69%) | 1584478.0(2.43%) | 4140148.0(6.34%) | 9423680.0(14.44%) | **7894490.0(12.1%)** |
| T96_4 | 36036841.0(62.46%) | 1535146.0(2.66%) | 3746621.0(6.49%) | 9749189.0(16.9%) | 6627749.0(11.49%) |

Table S2. Functional annotation statistics.

|  | Expre_Gene number (percent) | Expre_Transcript number (percent) | All_Gene number (percent) | All_Transcript number (percent) |
| --- | --- | --- | --- | --- |
| GO | 10914 (0.5052) | 22283 (0.4538) | 13543 (0.4264) | 27554 (0.4166) |
| KEGG | 9858 (0.4563) | 28578 (0.582) | 11176 (0.3519) | 33124 (0.5009) |
| EggNOG | 14646 (0.6779) | 38732 (0.7888) | 17420 (0.5485) | 46183 (0.6983) |
| NR | 17644 (0.8167) | 43933 (0.8947) | 22790 (0.7176) | 54539 (0.8247) |
| Swiss-Prot | 12818 (0.5933) | 35470 (0.7224) | 14793 (0.4658) | 41732 (0.631) |
| Pfam | 14104 (0.6528) | 37038 (0.7543) | 16517 (0.5201) | 43901 (0.6638) |
| Total_anno | 17962 (0.8314) | 44474 (0.9058) | 23405 (0.737) | 55414 (0.8379) |
| Total | 21605 (1.0) | 49101 (1.0) | 31758 (1) | 66134 (1) |

Table S3. Relative expression of viral genes detected in transcriptomic data from different time points of the *M. rosenbergii*.

| Seq _id | T24 | T48 | T72 | T96 | Subject sequence id | Identity (%) | ORF | Pridicted domains/function |
| --- | --- | --- | --- | --- | --- | --- | --- | --- |
| DIV1-ZH_00001 | 0.00 | 0.00 | 7141.23 | 1503.33 | YP_009552436.1 | 92.90 | 155L |  |
| DIV1-ZH_00002 | 0.00 | 16211.17 | 4470.13 | 4574.58 | YP_009552435.1 | 100.00 | 154R |  |
| DIV1-ZH_00003 | 0.00 | 0.00 | 1543.61 | 731.68 | YP_009552434.1 | 100.00 | 153R |  |
| DIV1-ZH_00004 | 0.00 | 0.00 | 275.03 | 6810.75 | YP_009552433.1 | 100.00 | 152R |  |
| DIV1-ZH_00005 | 0.00 | 0.00 | 14708.69 | 11300.24 | YP_009552432.1 | 100.00 | 151R |  |
| DIV1-ZH_00006 | 0.00 | 0.00 | 989.69 | 333.40 | YP_009552431.1 | 98.70 | 150R |  |
| DIV1-ZH_00007 | 0.00 | 0.00 | 39275.04 | 17440.92 | YP_009552429.1 | 100.00 | 148R |  |
| DIV1-ZH_00008 | 0.00 | 0.00 | 9958.34 | 8989.83 | YP_009552428.1 | 100.00 | 147R |  |
| DIV1-ZH_00009 | 0.00 | 56157.86 | 12378.54 | 879.92 | YP_009552427.1 | 100.00 | 146R |  |
| DIV1-ZH_00010 | 0.00 | 0.00 | 335.62 | 145.40 | YP_009552426.1 | 100.00 | 145R |  |
| DIV1-ZH_00011 | 0.00 | 0.00 | 3315.45 | 1323.45 | YP_009552425.1 | 99.60 | 144L |  |
| DIV1-ZH_00012 | 0.00 | 0.00 | 350.13 | 2994.18 | YP_009552424.1 | 100.00 | 143L |  |
| DIV1-ZH_00013 | 0.00 | 0.00 | 1059.80 | 0.00 | YP_009552423.1 | 100.00 | 142R |  |
| DIV1-ZH_00014 | 0.00 | 0.00 | 32290.65 | 26607.76 | YP_009552422.1 | 100.00 | 141L |  |
| DIV1-ZH_00015 | 0.00 | 0.00 | 25477.57 | 24138.88 | YP_009552421.1 | 100.00 | 140L |  |
| DIV1-ZH_00016 | 0.00 | 0.00 | 699.98 | 657.22 | UPA43879.1 | 99.20 | 139R | serine/threonine kinaseVRK1-like protein |
| DIV1-ZH_00017 | 0.00 | 0.00 | 1174.03 | 872.11 | UPA43717.1 | 99.40 | 138R |  |
| DIV1-ZH_00018 | 0.00 | 0.00 | 352.43 | 1556.19 | YP_009552418.1 | 100.00 | 137L |  |

Table S3. (continued)

| Seq _id | T24 | T48 | T72 | T96 | Subject sequence id | Identity (%) | ORF | Pridicted domains/function |
| --- | --- | --- | --- | --- | --- | --- | --- | --- |
| DIV1-ZH_00019 | 0.00 | 0.00 | 5368.52 | 4566.94 | YP_009552417.1 | 100.00 | 136R |  |
| DIV1-ZH_00021 | 0.00 | 0.00 | 0.00 | 774.04 | YP_009552415.1 | 100.00 | 134L |  |
| DIV1-ZH_00022 | 0.00 | 0.00 | 1076.54 | 667.38 | YP_009552414.1 | 100.00 | 133L |  |
| DIV1-ZH_00023 | 0.00 | 0.00 | 1364.71 | 768.76 | YP_009552413.1 | 100.00 | 132L |  |
| DIV1-ZH_00024 | 0.00 | 0.00 | 0.00 | 151.56 | YP_010084778.1 | 100.00 | 130R | thioredoxin2 |
| DIV1-ZH_00025 | 0.00 | 0.00 | 4335.73 | 492.99 | YP_009552410.1 | 100.00 | 129R |  |
| DIV1-ZH_00027 | 54338.84 | 10287.34 | 59399.40 | 76334.47 | UPA43707.1 | 100.00 | 127L | Ca2+-binding RTX toxin-related protein |
| DIV1-ZH_00028 | 0.00 | 0.00 | 389.05 | 592.13 | YP_009552407.1 | 99.50 | 126L |  |
| DIV1-ZH_00029 | 0.00 | 0.00 | 927.42 | 39.54 | YP_009552406.1 | 100.00 | 125R |  |
| DIV1-ZH_00032 | 0.00 | 0.00 | 6944.01 | 6519.81 | UPA43701.1 | 100.00 | 120L | Hypothetical protein 1DG000109 |
| DIV1-ZH_00033 | 0.00 | 0.00 | 16503.01 | 8194.10 | YP_009552400.1 | 100.00 | 119R |  |
| DIV1-ZH_00034 | 0.00 | 0.00 | 200.33 | 3590.26 | YP_009552399.1 | 99.50 | 118R |  |
| DIV1-ZH_00035 | 0.00 | 0.00 | 359.47 | 364.22 | YP_010084789.1 | 100.00 | 117R | Hypothetical protein KM509_gp037 |
| DIV1-ZH_00036 | 0.00 | 101836.58 | 986.62 | 202.80 | YP_009552397.1 | 100.00 | 116L |  |
| DIV1-ZH_00037 | 0.00 | 0.00 | 415.10 | 644.85 | UPA43425.1 | 100.00 | 115L | Hypothetical protein 4TH000151 |
| DIV1-ZH_00038 | 0.00 | 0.00 | 19400.56 | 14832.01 | YP_009552395.1 | 100.00 | 114L |  |
| DIV1-ZH_00039 | 0.00 | 0.00 | 7809.04 | 3812.92 | YP_009552394.1 | 100.00 | 113L |  |
| DIV1-ZH_00040 | 0.00 | 0.00 | 2804.19 | 789.09 | YP_010084794.1 | 100.00 | 112L | Hypothetical protein KM509_gp042 |

Table S3. (continued)

| Seq _id | T24 | T48 | T72 | T96 | Subject sequence id | Identity (%) | ORF | Pridicted domains/function |
| --- | --- | --- | --- | --- | --- | --- | --- | --- |
| DIV1-ZH_00041 | 0.00 | 0.00 | 0.00 | 95.64 | YP_009552392.1 | 100.00 | 111R |  |
| DIV1-ZH_00042 | 0.00 | 17389.21 | 570.06 | 4623.13 | YP_009552391.1 | 99.70 | 110R |  |
| DIV1-ZH_00043 | 0.00 | 0.00 | 8393.25 | 2308.87 | YP_009552390.1 | 99.20 | 109R |  |
| DIV1-ZH_00044 | 0.00 | 0.00 | 953.76 | 1343.63 | YP_009552389.1 | 94.90 | 108L |  |
| DIV1-ZH_00045 | 0.00 | 148163.42 | 13423.77 | 6908.44 | YP_010084799.1 | 99.20 | 107L | ribonuclease III |
| DIV1-ZH_00046 | 0.00 | 0.00 | 6415.77 | 3794.72 | YP_010084800.1 | 100.00 | 106R | Hypothetical protein KM509_gp048 |
| DIV1-ZH_00048 | 0.00 | 60411.05 | 6132.16 | 6156.26 | YP_009552386.1 | 100.00 |  | Head decoration |
| DIV1-ZH_00049 | 195661.16 | 0.00 | 2478.29 | 1821.59 | YP_010084802.1 | 100.00 | 104R | Hypothetical protein KM509_gp050 |
| DIV1-ZH_00050 | 0.00 | 0.00 | 636.93 | 879.53 | YP_009552384.1 | 99.80 | 103L |  |
| DIV1-ZH_00051 | 0.00 | 0.00 | 924.27 | 1172.44 | YP_009552383.1 | 100.00 | 102L |  |
| DIV1-ZH_00053 | 0.00 | 0.00 | 0.00 | 3137.99 | YP_009552381.1 | 100.00 | 100L |  |
| DIV1-ZH_00054 | 0.00 | 0.00 | 360.06 | 45.36 | YP_009552379.1 | 100.00 | 098L |  |
| DIV1-ZH_00055 | 0.00 | 261447.49 | 367.74 | 687.57 | YP_010084809.1 | 100.00 | 097L | DNA-dependent RNApolymeraseII largestsubunit |
| DIV1-ZH_00056 | 0.00 | 0.00 | 2382.90 | 2080.91 | YP_010084810.1 | 100.00 | 096L | Hypothetical protein KM509_gp058 |
| DIV1-ZH_00058 | 0.00 | 90113.60 | 252.25 | 3696.50 | YP_010084811.1 | 100.00 | 094R | Hypothetical protein KM509_gp059 |
| DIV1-ZH_00060 | 0.00 | 0.00 | 3332.44 | 4257.15 | UPA43677.1 | 100.00 | 092L | Hypothetical protein 1DG000085 |
| DIV1-ZH_00061 | 0.00 | 0.00 | 29962.92 | 8890.14 | YP_009552372.1 | 99.20 | 091R |  |

Table S3. (continued)

| Seq _id | T24 | T48 | T72 | T96 | Subject sequence id | Identity (%) | ORF | Pridicted domains/function |
| --- | --- | --- | --- | --- | --- | --- | --- | --- |
| DIV1-ZH_00062 | 0.00 | 0.00 | 197.22 | 268.76 | UPA43404.1 | 100.00 | 090R | Phytanoyl dioxygenase |
| DIV1-ZH_00063 | 0.00 | 0.00 | 1757.19 | 808.64 | YP_009552370.1 | 99.10 | 096L |  |
| DIV1-ZH_00064 | 0.00 | 0.00 | 1379.02 | 1946.97 | UPA43673.1 | 98.90 | 088L | NTPase helicase |
| DIV1-ZH_00065 | 0.00 | 0.00 | 138.36 | 527.99 | YP_009552368.1 | 100.00 | 087L | NAD-dependent DNAligase |
| DIV1-ZH_00066 | 0.00 | 0.00 | 7437.66 | 4916.18 | YP_009552367.1 | 96.80 | 086L |  |
| DIV1-ZH_00069 | 0.00 | 0.00 | 85.52 | 607.59 | UPA43398.1 | 100.00 | 084L | Hypothetical protein 4TH000124 |
| DIV1-ZH_00070 | 0.00 | 9088.11 | 3848.58 | 3833.33 | YP_010084821.1 | 100.00 | 083L | DNA-directed RNApolymeraseII second largest subunit |
| DIV1-ZH_00071 | 0.00 | 0.00 | 4368.35 | 3688.83 | UPA43396.1 | 99.80 | 082L | ATP-dependent helicase |
| DIV1-ZH_00072 | 0.00 | 34876.99 | 17722.16 | 12434.13 | YP_009552362.1 | 100.00 | 081R |  |
| DIV1-ZH_00073 | 0.00 | 0.00 | 5134.63 | 2281.14 | YP_009552361.1 | 99.40 |  | MutT/NUDIX hydrolase 160 |
| DIV1-ZH_00074 | 0.00 | 0.00 | 347.70 | 156.79 | YP_010084825.1 | 99.80 | 079L | Immediate early ICP-46 |
| DIV1-ZH_00075 | 0.00 | 0.00 | 2816.39 | 4875.64 | YP_009552359.1 | 100.00 | 078R |  |
| DIV1-ZH_00076 | 0.00 | 0.00 | 875.79 | 0.00 | YP_009552358.1 | 98.70 | 077L |  |
| DIV1-ZH_00077 | 0.00 | 0.00 | 13219.61 | 12100.21 | YP_009552357.1 | 100.00 | 076L |  |
| DIV1-ZH_00078 | 0.00 | 0.00 | 208.33 | 175.50 | UPA43390.1 | 99.90 |  | DNA topoisomerase II |
| DIV1-ZH_00079 | 0.00 | 0.00 | 197.08 | 179.24 | YP_010084830.1 | 99.30 | 074L | Hypothetical protein KM509_gp078 |
| DIV1-ZH_00080 | 0.00 | 0.00 | 3774.82 | 4248.14 | UPA43388.1 | 100.00 | 073R | Hypothetical protein 4TH000114 |
| DIV1-ZH_00081 | 0.00 | 0.00 | 5420.53 | 9133.47 | YP_010084832.1 | 100.00 | 072R | Hypothetical protein KM509_gp080 |

Table S3. (continued)

| Seq _id | T24 | T48 | T72 | T96 | Subject sequence id | Identity (%) | ORF | Pridicted domains/function |
| --- | --- | --- | --- | --- | --- | --- | --- | --- |
| DIV1-ZH_00082 | 0.00 | 0.00 | 0.00 | 806.88 | YP_009552352.1 | 100.00 | 071R |  |
| DIV1-ZH_00083 | 0.00 | 0.00 | 0.00 | 366.94 | YP_010084834.1 | 99.60 | 070R | Hypothetical protein KM509_gp082 |
| DIV1-ZH_00084 | 0.00 | 0.00 | 255922.83 | 326174.69 | YP_009552349.1 | 100.00 | 068R |  |
| DIV1-ZH_00085 | 0.00 | 0.00 | 1355.18 | 0.00 | YP_009552348.1 | 100.00 | 067R |  |
| DIV1-ZH_00086 | 0.00 | 0.00 | 2893.92 | 5943.83 | YP_009552347.1 | 100.00 | 066R |  |
| DIV1-ZH_00087 | 0.00 | 0.00 | 914.19 | 3053.52 | YP_009552346.1 | 100.00 | 065L |  |
| DIV1-ZH_00088 | 0.00 | 0.00 | 1019.12 | 960.12 | YP_009552345.1 | 98.60 | 064L |  |
| DIV1-ZH_00089 | 0.00 | 0.00 | 0.00 | 1041.67 | YP_009552344.1 | 100.00 | 063L |  |
| DIV1-ZH_00090 | 0.00 | 0.00 | 0.00 | 136.33 | UUT40566.1 | 61.40 |  | Hypothetical protein |
| DIV1-ZH_00093 | 0.00 | 0.00 | 0.00 | 415.66 | YP_009552343.1 | 100.00 | 062L |  |
| DIV1-ZH_00094 | 0.00 | 0.00 | 105.03 | 92.39 | YP_009552342.1 | 99.90 | 061L |  |
| DIV1-ZH_00095 | 0.00 | 0.00 | 2100.35 | 2521.88 | YP_009552341.1 | 100.00 | 060L |  |
| DIV1-ZH_00096 | 0.00 | 0.00 | 2451.56 | 2636.72 | YP_009552340.1 | 100.00 | 059L |  |
| DIV1-ZH_00097 | 0.00 | 0.00 | 3481.44 | 958.24 | YP_009552339.1 | 99.40 | 058L |  |
| DIV1-ZH_00098 | 0.00 | 0.00 | 918.58 | 2270.51 | YP_009552338.1 | 100.00 | 057R |  |
| DIV1-ZH_00099 | 0.00 | 0.00 | 1066.61 | 707.94 | YP_009552337.1 | 100.00 | 056R |  |
| DIV1-ZH_00100 | 0.00 | 0.00 | 214.46 | 3444.16 | YP_009552336.1 | 100.00 | 055L |  |
| DIV1-ZH_00101 | 0.00 | 0.00 | 2181.21 | 4392.48 | YP_009552335.1 | 100.00 | 054L |  |

Table S3. (continued)

| Seq _id | T24 | T48 | T72 | T96 | Subject sequence id | Identity (%) | ORF | Pridicted domains/function |
| --- | --- | --- | --- | --- | --- | --- | --- | --- |
| DIV1-ZH_00102 | 0.00 | 0.00 | 1636.09 | 4883.15 | YP_009552334.1 | 99.50 | 053L |  |
| DIV1-ZH_00103 | 0.00 | 0.00 | 5554.07 | 9460.91 | YP_010084851.1 | 93.10 | 052L | Hypothetical protein KM509_gp099 |
| DIV1-ZH_00104 | 0.00 | 0.00 | 0.00 | 107.91 | YP_010084852.1 | 99.60 | 051L | BRO-like protein |
| DIV1-ZH_00105 | 0.00 | 0.00 | 684.20 | 304.57 | YP_010084854.1 | 100.00 | 050L | Hypothetical protein KM509_gp102 |
| DIV1-ZH_00106 | 0.00 | 0.00 | 1820.21 | 3075.59 | YP_009552330.1 | 100.00 | 049R |  |
| DIV1-ZH_00107 | 0.00 | 0.00 | 1414.88 | 1185.54 | YP_009552329.1 | 100.00 | 048L |  |
| DIV1-ZH_00108 | 0.00 | 0.00 | 502.48 | 1522.24 | YP_009552328.1 | 100.00 | 047L |  |
| DIV1-ZH_00109 | 0.00 | 0.00 | 491.69 | 700.17 | YP_010084858.1 | 100.00 | 046L | Hypothetical protein KM509_gp106 |
| DIV1-ZH_00112 | 0.00 | 0.00 | 294.98 | 1722.56 | YP_009552324.1 | 100.00 | 043R |  |
| DIV1-ZH_00113 | 0.00 | 0.00 | 297.25 | 163.03 | YP_009552323.1 | 99.90 | 042L |  |
| DIV1-ZH_00115 | 0.00 | 0.00 | 6652.42 | 6405.64 | YP_009552321.1 | 100.00 | 040L |  |
| DIV1-ZH_00116 | 0.00 | 0.00 | 4977.99 | 3021.77 | YP_009552320.1 | 100.00 | 039R |  |
| DIV1-ZH_00117 | 0.00 | 0.00 | 461.46 | 208.82 | YP_009552319.1 | 99.50 | 038R |  |
| DIV1-ZH_00118 | 0.00 | 0.00 | 481.00 | 448.58 | YP_009552318.1 | 100.00 | 037L | DNA primase |
| DIV1-ZH_00119 | 0.00 | 0.00 | 3121.40 | 974.19 | YP_010084867.1 | 100.00 | 036R | Hypothetical protein KM509_gp115 |
| DIV1-ZH_00120 | 0.00 | 0.00 | 619.46 | 1328.31 | YP_009552316.1 | 100.00 | 035R |  |
| DIV1-ZH_00122 | 0.00 | 0.00 | 3906.99 | 843.83 | YP_010084870.1 | 99.60 | 033L | Hypothetical protein KM509_gp118, partial |
| DIV1-ZH_00123 | 0.00 | 0.00 | 1876.39 | 1423.51 | YP_009552313.1 | 100.00 | 032R |  |

Table S3. (continued)

| Seq _id | T24 | T48 | T72 | T96 | Subject sequence id | Identity (%) | ORF | Pridicted domains/function |
| --- | --- | --- | --- | --- | --- | --- | --- | --- |
| DIV1-ZH_00125 | 0.00 | 0.00 | 6816.83 | 1381.24 | YP_009552310.1 | 100.00 | 029L |  |
| DIV1-ZH_00126 | 0.00 | 0.00 | 47229.01 | 28468.87 | YP_009552309.1 | 100.00 | 028R |  |
| DIV1-ZH_00127 | 0.00 | 0.00 | 20863.05 | 16935.20 | UPA43617.1 | 100.00 | 027R | Hypothetical protein1 DG000025 |
| DIV1-ZH_00128 | 0.00 | 0.00 | 583.69 | 401.94 | UPA43616.1 | 99.50 | 026L | Cell surface protein |
| DIV1-ZH_00129 | 0.00 | 0.00 | 119.77 | 415.83 | YP_009552306.1 | 100.00 | 025L |  |
| DIV1-ZH_00130 | 0.00 | 0.00 | 0.00 | 11999.30 | YP_010084878.1 | 100.00 |  | Hypothetical protein KM509_gp126 |
| DIV1-ZH_00131 | 0.00 | 0.00 | 15826.51 | 30269.30 | YP_009552305.1 | 99.10 | 024L |  |
| DIV1-ZH_00132 | 0.00 | 0.00 | 23061.63 | 20669.84 | YP_009552304.1 | 97.50 | 023L |  |
| DIV1-ZH_00133 | 0.00 | 0.00 | 300.31 | 389.76 | YP_009552303.1 | 99.80 | 022R |  |
| DIV1-ZH_00134 | 0.00 | 0.00 | 1942.47 | 851.33 | YP_009552302.1 | 100.00 | 021L |  |
| DIV1-ZH_00135 | 0.00 | 0.00 | 2004.51 | 1026.27 | YP_009552301.1 | 99.50 | 020L |  |
| DIV1-ZH_00137 | 0.00 | 0.00 | 1659.36 | 5303.72 | YP_009552299.1 | 99.60 | 018R |  |
| DIV1-ZH_00138 | 0.00 | 0.00 | 1764.44 | 7341.88 | YP_009552298.1 | 100.00 | 017R |  |
| DIV1-ZH_00139 | 0.00 | 0.00 | 1360.72 | 105.38 | YP_009552297.1 | 100.00 | 016R |  |
| DIV1-ZH_00140 | 0.00 | 0.00 | 802.40 | 818.58 | YP_010084888.1 | 100.00 | 015R | N-acetylmuramoyl-L-alanineamidase |
| DIV1-ZH_00141 | 0.00 | 0.00 | 2348.30 | 749.57 | YP_009552295.1 | 100.00 | 014L |  |
| DIV1-ZH_00143 | 0.00 | 0.00 | 2086.11 | 119.78 | YP_009552293.1 | 100.00 | 012L |  |
| DIV1-ZH_00147 | 0.00 | 0.00 | 1962.58 | 3264.43 | YP_009552288.1 | 100.00 | 007L |  |

Table S3. (continued)

| Seq _id | T24 | T48 | T72 | T96 | Subject sequence id | Identity (%) | ORF | Pridicted domains/function |
| --- | --- | --- | --- | --- | --- | --- | --- | --- |
| DIV1-ZH_00148 | 0.00 | 0.00 | 1522.11 | 0.00 | YP_009552287.1 | 100.00 | 006R |  |
| DIV1-ZH_00149 | 0.00 | 34130.81 | 1701.66 | 7388.53 | YP_009552286.1 | 99.50 | 005R |  |
| DIV1-ZH_00150 | 0.00 | 0.00 | 216.33 | 88.60 | YP_009552285.1 | 100.00 | 004L |  |
| DIV1-ZH_00151 | 0.00 | 0.00 | 2647.73 | 1544.30 | YP_009552284.1 | 100.00 | 003L |  |
| DIV1-ZH_00153 | 0.00 | 0.00 | 3302.44 | 4249.27 | QUI88197.1 | 100.00 | 001R,partial | Major capsid protein, partial |
| DIV1-ZH_00154 | 0.00 | 88163.44 | 247.13 | 334.71 | YP_009552281.1 | 100.00 | 178R |  |
| DIV1-ZH_00155 | 0.00 | 36965.32 | 4258.40 | 10104.79 | YP_009552458.1 | 100.00 | 177L |  |
| DIV1-ZH_00156 | 0.00 | 0.00 | 4239.36 | 6817.03 | YP_009552457.1 | 100.00 | 176L |  |
| DIV1-ZH_00157 | 0.00 | 0.00 | 924.84 | 4256.29 | YP_010084904.1 | 100.00 | 175R | NTPase helicase |
| DIV1-ZH_00158 | 0.00 | 0.00 | 228.56 | 0.00 | YP_009552455.1 | 100.00 | 174R |  |
| DIV1-ZH_00159 | 0.00 | 0.00 | 1534.90 | 203.23 | YP_009552454.1 | 99.50 | 173R |  |
| DIV1-ZH_00160 | 0.00 | 0.00 | 1649.33 | 2273.67 | YP_009552453.1 | 100.00 | 172R |  |
| DIV1-ZH_00161 | 0.00 | 0.00 | 8565.47 | 3496.64 | YP_010084908.1 | 100.00 | 171L | Hypothetical protein KM509_gp156 |
| DIV1-ZH_00162 | 0.00 | 0.00 | 295.57 | 794.46 | YP_009552451.1 | 100.00 | 170R |  |
| DIV1-ZH_00163 | 0.00 | 0.00 | 64.45 | 89.66 | YP_009552450.1 | 100.00 | 169R |  |
| DIV1-ZH_00164 | 0.00 | 0.00 | 2588.15 | 1964.14 | YP_009552449.1 | 100.00 | 168L |  |
| DIV1-ZH_00165 | 0.00 | 0.00 | 5831.17 | 912.26 | YP_010084912.1 | 99.40 | 167L | Hypothetical protein KM509_gp160 |

Table S3. (continued)

| Seq _id | T24 | T48 | T72 | T96 | Subject sequence id | Identity (%) | ORF | Pridicted domains/function |
| --- | --- | --- | --- | --- | --- | --- | --- | --- |
| DIV1-ZH_00166 | 0.00 | 0.00 | 687.86 | 0.00 | UPA43310.1 | 100.00 | 166L | Hypothetical protein 4TH000036 |
| DIV1-ZH_00167 | 0.00 | 0.00 | 17965.76 | 16757.15 | UPA43309.1 | 100.00 | 165L | Hypothetical protein 4TH000035 |
| DIV1-ZH_00168 | 0.00 | 0.00 | 474.22 | 583.85 | YP_009552445.1 | 100.00 | 164L |  |
| DIV1-ZH_00169 | 0.00 | 0.00 | 461.74 | 0.00 | YP_010084916.1 | 100.00 | 163L | Appr-1-p processing protein |
| DIV1-ZH_00170 | 0.00 | 0.00 | 16477.80 | 11254.05 | YP_009552443.1 | 100.00 | 162R |  |
| DIV1-ZH_00171 | 0.00 | 0.00 | 21636.18 | 17893.61 | UPA43737.1 | 100.00 | 161R | Hypothetical protein1 DG000145 |
| DIV1-ZH_00172 | 0.00 | 0.00 | 4916.71 | 5803.80 | YP_009552441.1 | 100.00 | 160L |  |
| DIV1-ZH_00173 | 0.00 | 0.00 | 774.32 | 2205.04 | YP_009552440.1 | 99.70 | 159L |  |
| DIV1-ZH_00174 | 0.00 | 0.00 | 4969.05 | 3676.81 | YP_010084920.1 | 100.00 | 158R | Hypothetical protein KM509_gp168 |
| DIV1-ZH_00175 | 0.00 | 34757.65 | 14924.36 | 10822.36 | YP_010084921.1 | 99.40 | 157L | Myristylated membrane |
| DIV1-ZH_00176 | 0.00 | 0.00 | 7150.24 | 13989.26 | YP_010084922.1 | 100.00 | 156L | Hypothetical protein KM509_gp170 |

Table S4. DEGs enriched in three immune-related pathways.

| Gene name | Gene Description | CTRL48 | TR48 |
| --- | --- | --- | --- |
| **Lysosome-48h up** | |  |  |
| cathD | cathepsin D | 21.74 | 25.58 |
| LOC136828129 | lysosomal acid glucosylceramidase-like | 146.35 | 476.58 |
| LOC136828130 | lysosomal acid glucosylceramidase-like | 6.67 | 107.06 |
| LOC136834614 | sialin-like | 0.76 | 9.30 |
| LOC136845569 | sialin-like, transcript variant X2 | 0.29 | 3.16 |
| LOC136847721 | procathepsin L-like | 114.51 | 166.60 |
| LOC136825532 | beta-galactosidase-like | 53.95 | 102.54 |
| LOC136826203 | arylsulfatase B-like | 2.39 | 8.75 |
| LOC136826719 | N-sulphoglucosamine sulphohydrolase-like, transcript variant X4 | 9.39 | 18.15 |
| LOC136826720 | N-sulphoglucosamine sulphohydrolase-like | 2.81 | 11.26 |
| LOC136828513 | mite group 2 allergen Gly d 2.02-like | 20.18 | 106.16 |
| LOC136829317 | sphingomyelin phosphodiesterase-like | 129.18 | 169.58 |
| LOC136829434 | lysosomal alpha-mannosidase-like, transcript variant X1 | 51.83 | 173.07 |
| LOC136830133 | lipase 3-like, transcript variant X5 | 126.59 | 235.13 |
| LOC136831525 | arylsulfatase B-like, transcript variant X1 | 1.68 | 14.58 |
| LOC136831733 | arylsulfatase I-like | 10.75 | 24.31 |
| LOC136832512 | lysosomal protective protein-like | 6.80 | 10.35 |
| LOC136832602 | beta-hexosaminidase subunit alpha-like | 56.20 | 273.50 |
| LOC136836450 | beta-hexosaminidase subunit alpha-like, transcript variant X1 | 7.91 | 20.29 |
| LOC136833780 | putative inorganic phosphate cotransporter | 0.37 | 2.78 |
| LOC136834106 | alpha-N-acetylglucosaminidase-like | 43.93 | 194.15 |
| LOC136848155 | alpha-N-acetylgalactosaminidase-like, transcript variant X2 | 900.21 | 1481.08 |
| LOC136839165 | putative protein heh-1 | 0.81 | 4.81 |
| LOC136843953 | chitooligosaccharidolytic beta-N-acetylglucosaminidase-like | 316.49 | 748.96 |
| LOC136845959 | macrosialin-like | 95.50 | 355.69 |
| LOC136848855 | beta-galactosidase-like | 80.69 | 246.92 |
| LOC136849186 | lysosomal alpha-glucosidase-like | 1.84 | 2.87 |
| LOC136853634 | lysosomal acid glucosylceramidase-like | 82.98 | 181.17 |
| LOC136852937 | beta-glucuronidase-like, transcript variant X2 | 68.24 | 195.74 |
| LOC136854507 | sphingomyelin phosphodiesterase-like, transcript variant X1 | 39.03 | 65.23 |
| LOC136854922 | N-acetylgalactosamine-6-sulfatase-like | 79.28 | 124.65 |
| LOC136829222 | sphingomyelin phosphodiesterase-like isoform X2 | 19.64 | 55.22 |
| LOC136833916 | putative inorganic phosphate cotransporter | 0.68 | 2.45 |
| LOC136829248 | alpha-L-fucosidase-like | 38.98 | 215.08 |
| LOC136835564 | alpha-L-fucosidase-like | 147.67 | 661.94 |
| LOC136851882 | alpha-L-fucosidase-like isoform X2 | 12.88 | 56.21 |
| LOC136829247 | alpha-L-fucosidase-like, transcript variant X1 | 0.41 | 1.66 |
| LOC136832166 | alpha-L-fucosidase-like | 53.94 | 67.66 |

Table S4. (continued)

| Gene name | Gene Description | CTRL48 | TR48 |
| --- | --- | --- | --- |
| **down** |  |  |  |
| LOC136845005 | sialin-like | 40.91 | 5.18 |
| LOC136846904 | sialin-like | 126.23 | 20.06 |
| LOC136845510 | cathepsin B | 2.85 | 0.30 |
| LOC136845840 | cathepsin B-like | 267.85 | 48.28 |
| LOC136853983 | cathepsin L-like, transcript variant X1 | 122.51 | 22.01 |
| LOC136835439 | NPC intracellular cholesterol transporter 1-like | 90.06 | 15.71 |
| LOC136840798 | major facilitator superfamily domain-containing protein 8-like, transcript variant X3 | 97.40 | 20.98 |
| LOC136843206 | V-type proton ATPase 16 kDa proteolipid subunit c | 216.50 | 28.28 |
| VhaAC39-1 | V-type proton ATPase subunit VhaAC39-1 | 272.21 | 56.01 |
| VhaSFD | V-type proton ATPase subunit VhaSFD, transcript variant X1 | 285.54 | 54.01 |
| LOC136854070 | V-type proton ATPase subunit S1-like | 458.58 | 76.98 |
| VhaPPA1-1 | Vacuolar H[+] ATPase PPA1 subunit 1 | 468.92 | 111.59 |
| LOC136847181 | uncharacterized lncRNA | 15.69 | 2.66 |
| LOC136847207 | putative inorganic phosphate cotransporter, transcript variant X1 | 4.10 | 0.20 |
| LOC136849837 | crustapain-like | 5.24 | 0.47 |
| LOC136854076 | sphingomyelin phosphodiesterase-like | 31.13 | 2.92 |
| LOC136854969 | patched domain-containing protein 3-like | 21.79 | 0.01 |
| Rbcn-3B | WD repeat-containing protein Rbcn-3B, transcript variant X1 | 9.45 | 1.61 |
| LOC136833338 | leukocyte surface antigen CD53-like | 6.92 | 1.29 |
| LOC136832564 | lysosomal protective protein-like | 626.28 | 32.49 |
| LOC136831388 | digestive cysteine proteinase 2-like | 312.60 | 40.23 |
| LOC136830563 | chitooligosaccharidolytic beta-N-acetylglucosaminidase-like | 28.34 | 2.00 |
| LOC136826573 | patched domain-containing protein 3-like, transcript variant X1 | 144.32 | 1.54 |
| LOC136832568 | alpha-N-acetylgalactosaminidase-like, transcript variant X5 | 37.62 | 10.18 |
| LOC136826243 | putative inorganic phosphate cotransporter | 2.78 | 0.78 |
| LOC136836385 | putative triacylglycerol lipase | 39.11 | 1.57 |
| LOC136847487 | putative inorganic phosphate cotransporter isoform X3 | 2.01 | 0.27 |
| **Phagosome-48h up** | |  |  |
| LOC136833130 | tubulin alpha-1A chain-like | 0.24 | 4.41 |
| LOC136833131 | tubulin alpha-3 chain-like | 103.43 | 129.15 |
| LOC136840124 | tubulin beta-2B chain-like | 0.36 | 0.60 |
| LOC136838579 | integrin beta-PS-like, transcript variant X1 | 3.13 | 9.18 |
| LOC136843178 | integrin alpha pat-2-like | 0.57 | 1.80 |
| LOC136839005 | perlucin-like protein | 493.30 | 612.53 |
| LOC136853129 | perlucin-like protein | 0.15 | 4.32 |
| LOC136853134 | perlucin-like protein | 0.14 | 1.76 |
| LOC136840336 | perlucin-like protein, transcript variant X1 | 0.06 | 0.80 |

Table S4. (continued)

| Gene name | Gene Description | | | CTRL48 | | TR48 |
| --- | --- | --- | --- | --- | --- | --- |
| LOC136838699 | C-type lectin domain family 17, member A-like | | | 18.68 | | 42.00 |
| LOC136843733 | C-type lectin domain family 17, member A-like, transcript variant X1 | | | 81.08 | | 120.25 |
| LOC136845730 | integrin beta-PS-like, transcript variant X1 | | | 4.24 | | 13.74 |
| LOC136852391 | integrin alpha-8-like | | | 0.19 | | 0.44 |
| LOC136846519 | integrin beta pat-3-like | | | 6.19 | | 21.82 |
| LOC136845959 | macrosialin-like | | | 95.50 | | 355.69 |
| LOC136847721 | procathepsin L-like | | | 114.51 | | 166.60 |
| LOC136842151 | uncharacterized protein | | | 1.25 | | 3.26 |
| LOC136848218 | uncharacterized protein | | | 2.41 | | 8.44 |
| LOC136852387 | macrophage mannose receptor 1-like | | | 37.41 | | 57.73 |
| LOC136830831 | | actin-3-like, transcript variant X1 | 83.51 | | 282.62 | |
| LOC136855691 | actin, clone 403-like | | | 67.65 | | 175.38 |
| LOC136855692 | actin, clone 403-like | | | 47.08 | | 135.51 |
| LOC136827930 | CD209 antigen-like | | | 2.09 | | 13.78 |
| LOC136841385 | | protein croquemort-like, transcript variant X1 | 0.73 | | 2.17 | |
| LOC136853146 | hepatic lectin-like, transcript variant X1 | | | 0.12 | | 1.28 |
| Dhc64C | dynein heavy chain, cytoplasmic, transcript variant X1 | | | 13.22 | | 17.19 |
| **down** |  | | |  | |  |
| LOC136854070 | V-type proton ATPase subunit S1-like | | | 458.58 | | 76.98 |
| Vha13 | V-type proton ATPase subunit Vha13 | | | 1721.11 | | 399.56 |
| Vha14-1 | V-type proton ATPase subunit Vha14-1 | | | 801.60 | | 165.02 |
| Vha26 | V-type proton ATPase subunit Vha26 | | | 734.07 | | 145.81 |
| Vha44 | V-type proton ATPase subunit Vha44 | | | 334.57 | | 48.88 |
| VhaAC39-1 | V-type proton ATPase subunit VhaAC39-1 | | | 272.21 | | 56.01 |
| VhaPPA1-1 | Vacuolar H[+] ATPase PPA1 subunit 1 | | | 468.92 | | 111.59 |
| LOC136843206 | V-type proton ATPase 16 kDa proteolipid subunit c | | | 216.50 | | 28.28 |
| LOC136843265 | V-type proton ATPase subunit D-like | | | 424.19 | | 86.31 |
| VhaSFD | V-type proton ATPase subunit VhaSFD, transcript variant X1 | | | 285.54 | | 54.01 |
| LOC136855340 | protein peste-like, transcript variant X1 | | | 292.91 | | 0.04 |
| LOC136849837 | crustapain-like | | | 5.24 | | 0.47 |
| LOC136846805 | integumentary mucin A.1-like | | | 560.87 | | 133.19 |
| LOC136846387 | low affinity immunoglobulin epsilon Fc receptor-like | | | 1.90 | | 0.18 |
| LOC136831388 | digestive cysteine proteinase 2-like | | | 312.60 | | 40.23 |
| **C-type lectin receptor signaling pathway-48h up** | | | |  | |  |
| Ras64B | ras-like protein 2 | | | 0.58 | | 1.83 |
| LOC136827930 | CD209 antigen-like | | | 2.09 | | 13.78 |
| LOC136833350 | tyrosine-protein kinase Fyn-like | | | 0.55 | | 1.45 |
| LOC136838699 | C-type lectin domain family 17, member A-like | | | 18.68 | | 42.00 |
| LOC136843733 | C-type lectin domain family 17, member A-like | | | 81.08 | | 120.25 |

Table S4. (continued)

| Gene name | Gene Description | CTRL48 | TR48 |
| --- | --- | --- | --- |
| LOC136839005 | perlucin-like protein | 493.30 | 612.53 |
| LOC136840336 | perlucin-like protein, transcript variant X1 | 0.06 | 0.80 |
| LOC136853129 | perlucin-like protein | 0.15 | 4.32 |
| LOC136853134 | perlucin-like protein | 0.14 | 1.76 |
| LOC136853136 | perlucin-like protein, transcript variant X1 | 0.19 | 4.30 |
| LOC136853142 | perlucin-like protein, transcript variant X1 | 0.44 | 3.52 |
| LOC136853150 | perlucin-like protein, transcript variant X1 | 0.09 | 1.45 |
| LOC136843190 | prostaglandin G/H synthase 2-like | 2.29 | 6.78 |
| LOC136854583 | prostaglandin G/H synthase 1-like | 0.38 | 0.87 |
| LOC136856198 | uncharacterized protein, transcript variant X1 | 10.52 | 14.20 |
| LOC136853146 | hepatic lectin-like, transcript variant X1 | 0.12 | 1.28 |
| Pi3K21B | phosphatidylinositol 3-kinase regulatory subunit alpha | 12.26 | 15.56 |
| **down** |  |  |  |
| LOC136853610 | NF-kappa-B inhibitor cactus-like, transcript variant X1 | 57.02 | 10.69 |
| LOC136856620 | uncharacterized protein | 55.68 | 9.04 |
